# A detailed view of the intracellular transcriptome of *Listeria monocytogenes* in murine macrophages using RNA-seq

**DOI:** 10.3389/fmicb.2015.01199

**Published:** 2015-10-30

**Authors:** Tilman Schultze, Rolf Hilker, Gopala K. Mannala, Katrin Gentil, Markus Weigel, Neda Farmani, Anita C. Windhorst, Alexander Goesmann, Trinad Chakraborty, Torsten Hain

**Affiliations:** ^1^Institute of Medical Microbiology, Justus Liebig UniversityGiessen, Germany; ^2^Bioinformatics and Systems Biology, Justus Liebig UniversityGiessen, Germany; ^3^Institute of Medical Informatics, Justus Liebig UniversityGiessen, Germany

**Keywords:** *Listeria monocytogenes*, intracellular, mRNA transcriptome analysis, RNA-seq, human pathogenic bacteria

## Abstract

*Listeria monocytogenes* is a bacterial pathogen and causative agent for the foodborne infection listeriosis, which is mainly a threat for pregnant, elderly, or immunocompromised individuals. Due to its ability to invade and colonize diverse eukaryotic cell types including cells from invertebrates, *L. monocytogenes* has become a well-established model organism for intracellular growth. Almost 10 years ago, we and others presented the first whole-genome microarray-based intracellular transcriptome of *L. monocytogenes*. With the advent of newer technologies addressing transcriptomes in greater detail, we revisit this work, and analyze the intracellular transcriptome of *L. monocytogenes* during growth in murine macrophages using a deep sequencing based approach. We detected 656 differentially expressed genes of which 367 were upregulated during intracellular growth in macrophages compared to extracellular growth in Brain Heart Infusion broth. This study confirmed ∼64% of all regulated genes previously identified by microarray analysis. Many of the regulated genes that were detected in the current study involve transporters for various metals, ions as well as complex sugars such as mannose. We also report changes in antisense transcription, especially upregulations during intracellular bacterial survival. A notable finding was the detection of regulatory changes for a subset of temperate A118-like prophage genes, thereby shedding light on the transcriptional profile of this bacteriophage during intracellular growth. In total, our study provides an updated genome-wide view of the transcriptional landscape of *L. monocytogenes* during intracellular growth and represents a rich resource for future detailed analysis.

## Introduction

*Listeria monocytogenes* is a ubiquitously distributed pathogen. It grows under a variety of adverse conditions like high salt concentration or low temperatures thereby resisting many common food preservation techniques. *L. monocytogenes* contaminated food is a serious health threat and mainly immunocompromised individuals, elderly persons and pregnant women suffer from severe complications (Hamon et al., 2006; Allerberger and Wagner, 2010; Cossart and Lebreton, 2014; Elinav et al., 2014). Upon ingestion of contaminated food, *L. monocytogenes* reaches the gastrointestinal tract where it invades epithelial cells. Via infection of macrophages and escape from their phagosome, *L. monocytogenes* ultimately reaches liver and spleen. Due to its versatile ability of infecting mammalian and invertebrate cells, *L. monocytogenes* has become a popular model organism for intracellular pathogenicity (Hamon et al., 2006; Lecuit, 2007; Cossart and Toledo-Arana, 2008; Cossart, 2011).

Recently, non-coding RNA elements were frequently studied in *L. monocytogenes* (Mellin and Cossart, 2012). Non-coding RNA elements are widely implicated in adaptation to different environmental conditions, metabolism, and virulence. The role of regulatory RNA elements (Johansson et al., 2002; Mansjö and Johansson, 2011), small RNA (Christiansen et al., 2006; Mandin et al., 2007; Nielsen et al., 2008; Oliver et al., 2009; Toledo-Arana et al., 2009; Mraheil et al., 2011; Nielsen et al., 2011; Sievers et al., 2014, 2015) and antisense RNA (Toledo-Arana et al., 2009; Wurtzel et al., 2012; Behrens et al., 2014; Wehner et al., 2014) was investigated. A new class of regulatory elements, the excludon was defined as long transcript that function as both sense transcript for one gene (or set of genes) as well as antisense transcript for a divergently oriented neighboring gene (or set of genes; Wurtzel et al., 2012).

Intracellular growth of *L. monocytogenes* was included as experimental condition in several genome-wide transcriptional profiling studies, however, few publications have focused on the changes in the mRNA transcriptome. These studies were conducted under very diverse conditions, making comparisons between studies difficult. The mRNA transcriptional profile of intracellular *L. monocytogenes* 6 h after infection of Caco-2-ephithelial cells was assessed by DNA microarray analysis (Joseph et al., 2006). In this study – as in every other that followed – the transcriptome of *L. monocytogenes* grown in rich media Brain Heart Infusion (BHI) served as reference. A similar approach was used to examine the transcriptome of *L. monocytogenes* when grown in murine macrophages P388D1 (Chatterjee et al., 2006). Here, the transcriptome of bacteria after 4 and 8 h post-infection as well as during intravacuolar growth was analyzed. Camejo et al. (2009) introduced *in vivo* work that shed light on the transcriptional profile of *L. monocytogenes* in murine spleens at 24, 48, and 72 h post-intravenous infection.

Despite the introduction of *next generation sequencing* (NGS) technologies, no study has addressed transcriptional changes in *L. monocytogenes* in detail. We now revisit the earlier microarray-based work (Chatterjee et al., 2006) complementing it with recent RNA-seq data (Wehner et al., 2014) which exclusively investigated non-coding RNAs of *L. monocytogenes* under intra- and extracellular growth conditions.

Here, we report the transcriptional changes in *L. monocytogenes* during intracellular growth in murine macrophages using an approach that also detects alteration of non-coding RNA elements. Furthermore, we construct a framework for further analysis targeting intracellular survival of *L. monocytogenes*. Finally, we outline common transcriptional responses of *L. monocytogenes* during intracellular growth among all the diverse growth conditions investigated to date.

## Materials and Methods

### Bacterial Strains, Plasmids, and Culture Conditions

All bacterial strains and plasmids used in this study are listed in **Table 1.** Wild type *L. monocytogenes* EGD-e serotype 1/2a (Glaser et al., 2001) and its isogenic deletion mutants Δ*lmo2316* or Δ*lmo1119* were cultivated under aerobic conditions in BHI broth (Difco, Becton Dickinson, Franklin Lakes, NJ, USA) or on BHI agar plates at 37°C. For RNA-seq experiments, *L. monocytogenes* EGD-e was grown in BHI broth overnight at 37°C with shaking at 180 rpm. Bacterial cultures were supplemented with fresh medium and grown until OD_600_ reached 1.0.

**Table 1:** Bacterial strains and plasmids used in this study.

Strain or plasmid	Description	Reference
*Listeria monocytogenes* EGD-e	Wild type	Glaser et al., 2001
DH10β	Electrocompetent	Life technologies, Carlsbad, CA, USA
Δ*lmo1119*	*lmo1119* isogenic deletion mutant	This study
Δ*lmo2316*	*lmo2316* isogenic deletion mutant	This study
Δ*lmo1119/lmo2316*	*lmo1119 and lmo2316* isogenic double deletion mutant	This study
pCR2.1-TOPO	single 3′-thymidine (T) overhangs for TA Cloning^®^ 3.9 kb	Life technologies, Carlsbad, CA, USA
pAUL-A	Temperature sensitive shuttle vector 9.2 kb	Schäferkordt and Chakraborty, 1995


*Escherichia coli* strain (DH10β) was grown in Luria-Bertani (LB) broth or on LB agar plates at 37°C. Cultures were supplemented with 300 μg/ml erythromycin (Sigma-Aldrich, St. Louis, MO, USA) for *E. coli* and 5 or 10 μg/ml for erythromycin for *L. monocytogenes* or 50 μg/ml ampicillin (Sigma-Aldrich, St. Louis, MO, USA) for *E. coli* and 200 μg/ml ampicillin for *L. monocytogenes*.

### Construction of Chromosomal Deletion Mutants Δ*lmo2316*, Δ*lmo1119*, and Δ*lmo1119/lmo2316*

Generation of *lmo2316 or lmo1119* in frame deletion mutants was performed as previously described (Seifart Gomes et al., 2011). A detailed description is provided as **Supplemental Text 1.**

### RNA Isolation and RNA Sequencing

Bacterial RNA isolation was carried out as described previously (Wehner et al., 2014). The quality of total RNA was assessed using Bioanalyzer 2100 Total RNA nano chip (Agilent, Santa Clara, CA, USA) and RNA concentration was measured using Qubit 2.0 fluorimeter (Invitrogen/Life Technologies, Carlsbad, CA, USA). rRNA was depleted from 1 μg of total RNA using Ribo-Zero Magnetic Kit (Bacteria; Epicentre, Madison, WI, USA). Depleted RNA was then treated with tobacco acid pyrophosphatase (Epicentre, Madison, WI, USA) and cleaned up with the RiboMinus concentration module (Life Technologies, Carlsbad, CA, USA).

For fragmentation and further library preparation TruSeq Stranded Total RNA Seq Kit (Illumina, San Diego, CA, USA) was used according to manufacturer’s instruction. In this process a barcoded-approach was chosen to facilitate multiplexed sequencing. For reverse transcription Superscript II polymerase (Life Technologies, Carlsbad, CA, USA) was used and libraries were purified with AMPure XP Reagent (Beckman Coulter, Pasadena, CA, USA). The yield and size distribution of the amplified cDNA were assessed with DNA High sensitivity kit (Agilent, Santa Clara, CA, USA). Libraries were then diluted to 4 nM, pooled, denatured and further diluted to 10 pM. Sequencing was carried out on the MiSeq using v2 chemistry (Illumina, San Diego, CA, USA).

### NGS Data Analysis

FastQ-files of sequencing runs performed with Ion Torrent PGM (Wehner et al., 2014) were downloaded from EBI (accession number PRJEB6949).

FastQ-files for MiSeq runs were obtained after image processing, basecalling and demultiplexing of reads performed by Illumina MiSeq-Control Software version 2.1.13 using RTA-version 1.17.22. For online accessibility, fastQ-files of the MiSeq runs were stored at the European Nucleotide Archive (accession number PRJEB10393).

Sequencing reads were mapped against *L. monocytogenes* EGD-e (NCBI RefSeq NC_003210.1). Reads were aligned with bowtie2 version 2.1 (Langmead and Salzberg, 2012). Read type was set to single-end for all runs. SAM-files of all PGM runs were used as input for read counting using HTSeq count version 0.6.1 (Anders et al., 2014). Strand-specificity was taken into account and rRNA (lmor), tRNA (lmot) as well as small RNA (lmos or former rli) were excluded from counting. The default counting mode ‘union’ was selected. All differential expression analyses were performed in R version 3.1.1 R Core Team (2014) using the package DESeq2 (Love et al., 2014). *p*-values were adjusted by the default method in DESeq2 and genes were considered differentially expressed when adjusted *p*-values below 0.05 were obtained.

While only the PGM runs were used for the differential expression model, all visualizations of read mappings and coverage plots were generated from the additional experiments run on the Illumina MiSeq. 3,556,623 reads aligned to the reference exactly one time for the extracellular condition and 6,503,703 reads aligned for the intracellular condition. The difference was caused by variances in sequencing output, as mapping rates were alike for both experiments (75 and 79%). Visualizations were then created using ReadXplorer version 2.0.1. (Hilker et al., 2014).

### Quantitative Real-Time PCR

mRNA levels of *lmo1119* and *lmo2316* from both extra- and intracellularly grown bacteria were assessed using quantitative real-time-PCR (qRT-PCR) as previously described (Wehner et al., 2014). Each qRT-PCR was performed in triplicate (locus tag _5′ and locus tag _3′, **Supplementary Table S1**; 7900 HT Fast Real Time System, Applied Biosystems/Thermo Fisher Scientific, Waltham, MA, USA). Gene expression was normalized to 16S rRNA and relative expression of *lmo1119* and *lmo2316* was calculated using the mathematical method published by Pfaﬄ and Pfaﬄ (2001).

### *In vitro* Infection

P388D1 murine macrophage were infected with *L. monocytogenes* EGD-e and its isogenic deletion mutants as described previously (Mraheil et al., 2011).

### Statistical Analysis for qRT-PCR and Survival Assays Results

All experimental work was repeated for a minimum of three times. Statistical analyses for qRT-PCR and survival assays were performed using R version 3.1.3. Data was log-transformed. Differential expression detected by qRT-PCR was tested by Student’s one-sample *t*-test. Differences in survival were analyzed using a linear regression model (Chambers and Hastie, 1992), where the amount of colony forming units (CFUs) was corrected by the inoculation amount as covariable. Pairwise comparisons were performed using the R package lsmeans version 2.17^1^ and multcomp (Hothorn et al., 2008). *p* < 0.05 was considered significant.

## Results

We based our work on a recent study which included six sequencing runs (three runs for extracellular and three runs for intracellular growth conditions) performed with IonTorrent semiconductor sequencing technology (Wehner et al., 2014). This study investigated regulatory RNA, changes of mRNA expression were beyond the scope of the work.

To extend and further back up this data set additional experiments were included using the reversible dye-terminator based sequencing approach of the Illumina MiSeq. These additional runs also facilitate detailed analysis of read mappings due to a higher amount of mapped reads and thus higher overall depth of coverage. In addition, it enables the evaluation whether transcriptomic changes occur platform-independently. Overall, pairwise comparison of normalized read counts provided no indication that variances in read counts between Illumina and Ion torrent runs exceeded variances of biological repeats among the Ion torrent runs (data not shown).

### Intracellular mRNA Transcriptome of *L. monocytogenes*

Comparing extracellular to intracellular growth conditions, our results show differential regulation for 656 genes (nearly 23% of all genes).The genes with the most dramatic changes in expression are listed in **Table 2.**

**Table 2:** Except of differentially expressed genes under intracellular growth conditions compared to extracellular growth.

Locus tag	Gene	Description	Regulation (intracellular)
*lmo0205*	*plcB*	Phospholipase C	Up
*lmo0203*	*mpl*	Zinc metalloproteinase precursor	Up
*lmo2751*	*lmo2751*	ABC transporter ATP-binding protein	Up
*lmo0204*	*actA*	Actin-assembly inducing protein precursor	Up
*lmo1786*	*inlC*	Internalin C	Up
*lmo0202*	*hly*	Listeriolysin O precursor	Up
*lmo0207*	*lmo0207*	Hypothetical protein	Up
*lmo0838*	*hpt*	Sugar:phosphate antiporter	Up
*lmo0608*	*lmo0608*	ABC transporter ATP-binding protein	Up
*lmo2828*	*lmo2828*	Hypothetical protein	Up
*lmo0206*	*lmo0206*	Hypothetical protein	Up
*lmo0751*	*lmo0751*	Hypothetical protein	Up
*lmo2752*	*lmo2752*	ABC transporter ATP-binding protein	Up
*lmo0576*	*lmo0576*	Hypothetical protein	Up
*lmo0955*	*lmo0955*	Hypothetical protein	Up
*lmo0514*	*lmo0514*	Internalin	Up
*lmo0749*	*lmo0749*	Hypothetical protein	Up
*lmo2102*	*lmo2102*	Glutamine amidotransferase subunit PdxT	Down
*lmo0954*	*lmo0954*	Hypothetical protein	Up
*lmo0748*	*lmo0748*	Hypothetical protein	Up
*lmo0750*	*lmo0750*	Hypothetical protein	Up
*lmo2827*	*lmo2827*	MarR family transcriptional regulator	Up
*lmo0607*	*lmo0607*	ABC transporter ATP-binding protein	Up
*lmo0201*	*plcA*	Phosphatidylinositol-specific phospholipase C	Up


*Genes ordered descendingly by p-value.*

Moreover, **Supplementary Table S2** provides a complete list of all genes that were differentially expressed under intracellular growth conditions. Of those 656 genes, 367 were predicted to be up- and 289 to be downregulated when grown in murine macrophages. These genes were further characterized using the previously introduced operon model (Toledo-Arana et al., 2009). Among the 656 differentially regulated genes, 386 are organized in operon structures while 270 are transcribed monocistronically. Further analysis of the differential expression pattern of these operons is given in **Supplemental Text 2** as well as **Supplementary Figure S1.**

### Concordance of RNA-seq Results with Microarray Data

When comparing our current results to the results of the previous microarray approach, we confirmed 55% of all differentially expressed genes found by the microarray-based approach. In both cases, an arbitrary threshold (*p*-value below 0.05) was applied to distinguish between differentially and not differentially expressed genes. A threshold of *p* < 0.1 in the current analysis increases the number to 64% of the previously found genes.

Five differentially regulated genes show opposing directions of regulation in both studies. These genes, one hypothetical gene as well as the metal transport operons *lmo1423–1424* and *lmo1848–1849*, suggest that there have been minor experimental differences between both experimental conditions.

**FIGURE 1:**
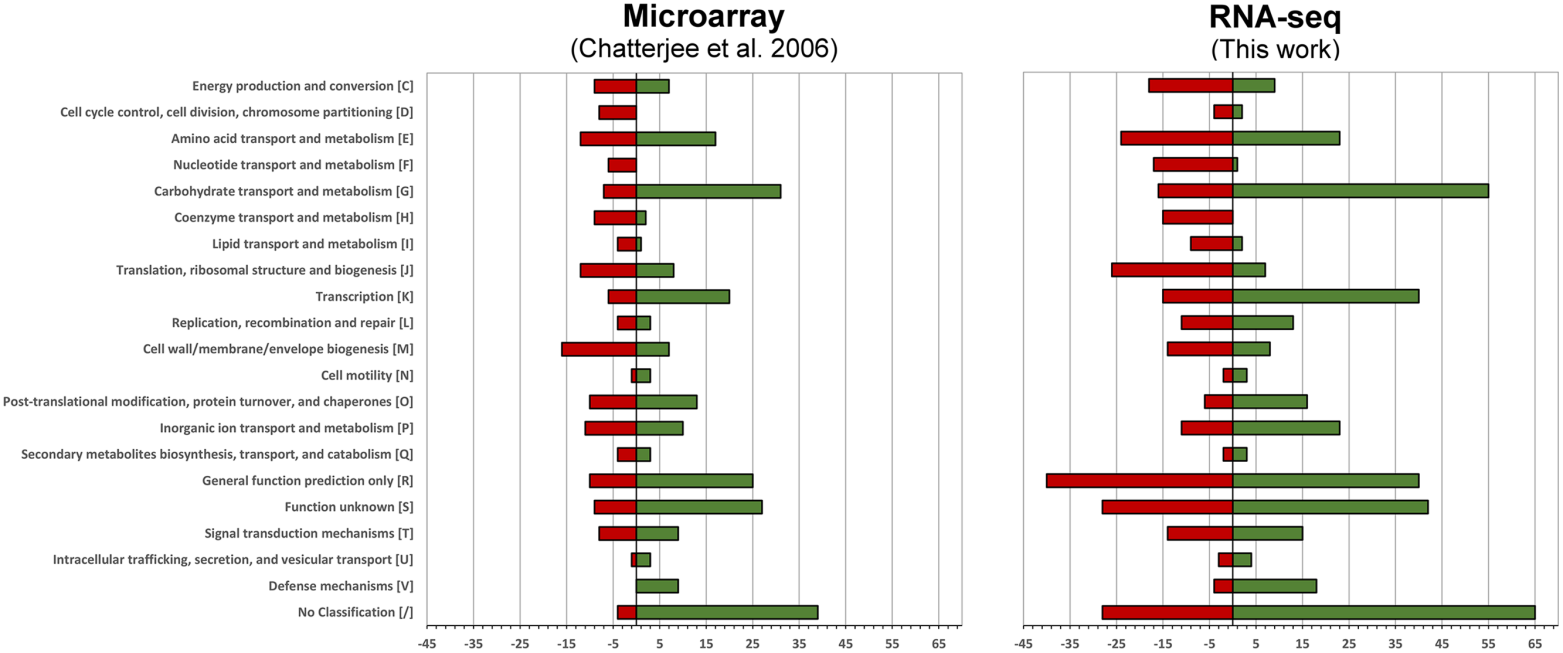
**Functional changes in response to intracellular growth conditions based on Cluster of Orthologous Groups of proteins (COG) classification.** Shown are the functional changes for the current RNA-seq approach **(right)** and the earlier microarray-based work **(left)**. For each class the number of differentially regulated genes among this class is visualized by a bar. Red bars represent the downregulated and green bars the upregulated genes when grown in murine macrophages.

We then compared how the differentially expressed genes translate into alterations of functional pathways by applying the Clusters of Orthologous Groups of proteins (COG)^2^ classification. The number of differentially expressed genes is higher in the current RNA-seq study, however, the effects on the regulation of functional pathways is very similar (**Figure 1**).

### Core Set of Genes Essential for Intracellular Growth

The investigated intracellular growth conditions vary widely among all published studies (Chatterjee et al., 2006; Joseph et al., 2006; Camejo et al., 2009; Toledo-Arana et al., 2009). We identified common transcriptional responses among these studies and our current work that are described in detail.

#### Virulence Genes are Upregulated during Intracellular Growth

Regardless whether epithelial cells, macrophages, blood, intestine, or spleens were infected, upregulation of the gene cluster *lmo0201–0205* was reported under intracellular growth conditions. Genes encoded by this virulence gene cluster are *actA* (intracellular motility), metalloprotease (*mpl*), two phospholipases (*plcA*, *plcB*), and the pore forming toxin listeriolysin (*hly*). The virulence gene cluster is controlled by the positive regulatory factor PrfA. *prfA* was consistently upregulated during intracellular growth in all studies. In addition to the virulence cluster, PrfA-regulated expression has been reported for *lmo0207* (*orfZ*), *lmo0796*, *lmo0434* (*inlB*), *lmo1786* (*inlC*), *lmo2219* (*prsA2*), and *lmo0838* (*hpt*). *lmo0207* and *lmo0206*, which were significantly upregulated in all studies except one (Camejo et al., 2009), are genes of poorly understood function adjacent to the virulence cluster. *lmo0434* (*inlB*) and *lmo1786* (*inlC*) encoding internalins needed for invasion as well as *lmo2219* (*prsA2*) encoding a predicted peptidyl-prolyl isomerase. The PrfA-regulated gene *lmo0838* is involved in the transport of phosphorylated sugars. Apart from PrfA-regulated genes, the studies by Camejo, Chatterjee as well as our current study report upregulation of four VirR-regulated genes. Two of these genes are encoding hypothetical proteins (*lmo0604* and *lmo2177)* and two are encoding ABC transporter *(lmo2114* and *lmo2115)*.

#### Stress-response Genes are Induced during Intracellular Growth

Upregulation of genes involved in stress response represents a second principal finding. This mechanism was found for *L. monocytogenes* grown in epithelial cells, macrophages, splenocytes, and bacteria grown in the intestine (Chatterjee et al., 2006; Joseph et al., 2006; Camejo et al., 2009; Toledo-Arana et al., 2009) Specifically, the upregulation of *lmo1473 (dnaK), lmo1474 (grpE)*, and *lmo2068* (*groEL*) belonging to heat shock molecular chaperons (class I) as well as *lmo2206* (*clpB*) which is an ATP-dependent protease (class III), was found in all studies. In addition to the induced transcription of *lmo1473* and *lmo1474*, upregulation of *lmo1471* (*prmA*), *lmo1472* (*dnaJ*) and *lmo1475* (*hrcA*), being part of the same operon, was found by our study as well as by the previous microarray-approach. Furthermore, our study suggests a broader transcriptional upregulation for class III heat shock genes. Transcripts of the whole major heat shock class III operon consisting of *lmo0229* to *lmo0232* were significantly increased. Especially, *lmo0232* (*clpC*) was significantly upregulated in all studies except the one using epithelial cells as host. Similarly, the caseinolytic protease proteins *lmo0997* (*clpE*) was upregulated in splenocytes and macrophages.

#### Metabolic Pathways are Differentially Regulated during Intracellular Growth

The last common finding among all studies concerns the intracellular transcriptional upregulation for *lmo1538*. *lmo1538* encodes a glycerol kinase that is required for intracellular growth. Regulation of glycolysis under intracellular growth conditions was also addressed by the previous studies. Central genes of glycolysis were upregulated when *L. monocytogenes* was grown in splenocytes (Camejo et al., 2009), the studies in epithelial cells and macrophages found downregulation (Chatterjee et al., 2006; Joseph et al., 2006). For instance, it was observed that the operon *lmo2455–2460*, encoding five essential enzymes for the terminal glycolysis steps, has been downregulated.

### Novel Findings of this Study

Additional metabolic changes extending previous findings involve the transport of sugar and sugar phosphates. Our results provide evidence for the downregulation of mannose transporter subunits *lmo0096–0098* as well as *lmo1389–1390*, genes involved in sugar transport. In contrast, upregulation was observed for *lmo0736–0739*, contributing to sugar transport and processing of phosphorylated ribulose, and an entire operon (*lmo2733–2736*), encoding fructose transporter, sugar hydrolase and sucrose phosphorylase. Remarkably, the operon *lmo2670–2672* was also uniformly upregulated during intracellular growth, including *lmo2671*, a hypothetical protein that has similarity to a lactoylglutathione lyase. Genes required for phosphate transport were also differentially expressed. One of those loci consists of the genes *lmo2494–2499*, encoding proteins for phosphate uptake and transport. In addition, operon *lmo2248* (a putative phosphate transport regulator)/*lmo2249* (a low-affinity inorganic phosphate transporter) was downregulated during intracellular growth. Finally, several genes involved in DNA repair, e.g., *recA* (*lmo1398*) and the excinucleases *lmo2050*, *lmo2488* (*uvrA*)/*lmo2489* (*uvrB*) were differentially expressed during intracellular growth conditions.

#### Antisense Transcription is Altered during Intracellular Growth

A benefit of strand-specific RNA-seq studies is the ability to detect antisense transcripts as well as sense transcripts. We investigated changes in antisense expression between intra- and extracellular growth conditions. These analyses were not possible with previous microarray based approaches.

For many of the genes showing dramatic differential antisense expression during intracellular growth, adjacent divergent genes displayed drastic changes in sense expression. Examples are antisense transcripts against *lmo0208–0209* and *lmo0198–0199* both flanking the virulence gene cluster. Similarly, antisense transcripts of *lmo0839* (neighboring the divergently oriented *hpt*) and of the sugar transferase gene *lmo0497* (adjacent a highly upregulated SOS response gene *lmo0496*), were highly induced.

**FIGURE 2:**
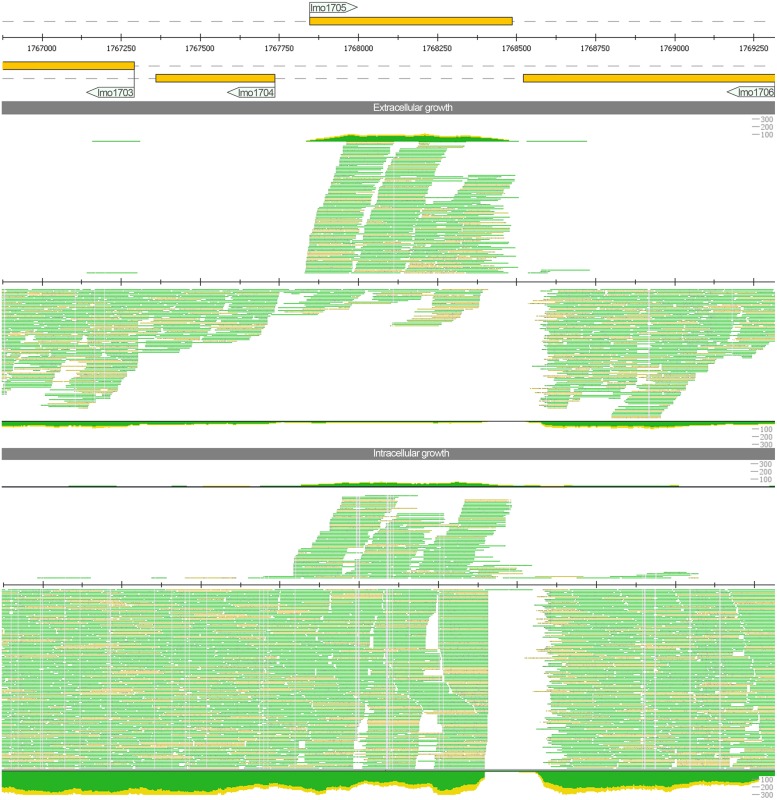
**Read mapping for the locus *lmo1705* under extracellular and intracellular growth conditions generated with ReadXplorer (Hilker et al., 2014).** The three tracks represent genome annotation of this locus, read mapping for extra- and for intracellular conditions (in top–down order). For each track, genes on the plus strand (respectively their transcripts) are depicted above the central line, while minus strand genes (or their transcripts) are shown below. For the two read mapping tracks (extra- and intracellular growth condition), a detailed alignment up to a depth of 35 reads and a coverage plot is provided, too. The color thereby indicates whether reads are uniquely mapped and match the reference perfectly (green) or whether they map uniquely, but with mismatches (yellow). The read mapping indicates a strong increase in antisense transcripts for *lmo1705* during intracellular growth.

We also detect strong antisense upregulation for *lmo1705* that was previously reported only as marginal note in the supplement (Wehner et al., 2014). With the additional sequencing runs that provided a higher read coverage, we obtained a distinct picture. Sense transcripts are slightly downregulated during intracellular growth while antisense transcripts are upregulated. When analyzing the read pattern (**Figure 2**) hardly any read was spanning the intergenic region between *lmo1705* and *lmo1706.* Hence, the increase of antisense reads could not be attributed to an erroneous lack of transcription termination of the adjacent *lmo1706*. Moreover, a high coverage was found for the intergenic region between *lmo1705* and *lmo1704*. Operon *lmo1702–1704* appears to have an additional 5′ UTR transcription start site, which overlaps *lmo1705*. The complete list with genes for which putative changes in antisense expression were found is provided in **Supplementary Table S3**.

#### Regulation of the A118-like Prophage Genes in Intracellular Conditions

In our study, we found increased expression of a cassette of A118-like prophage genes (*lmo2271*–*2332*) under intracellular growth conditions. In contrast, only few genes were predicted to be upregulated by the previous studies.

**FIGURE 3:**
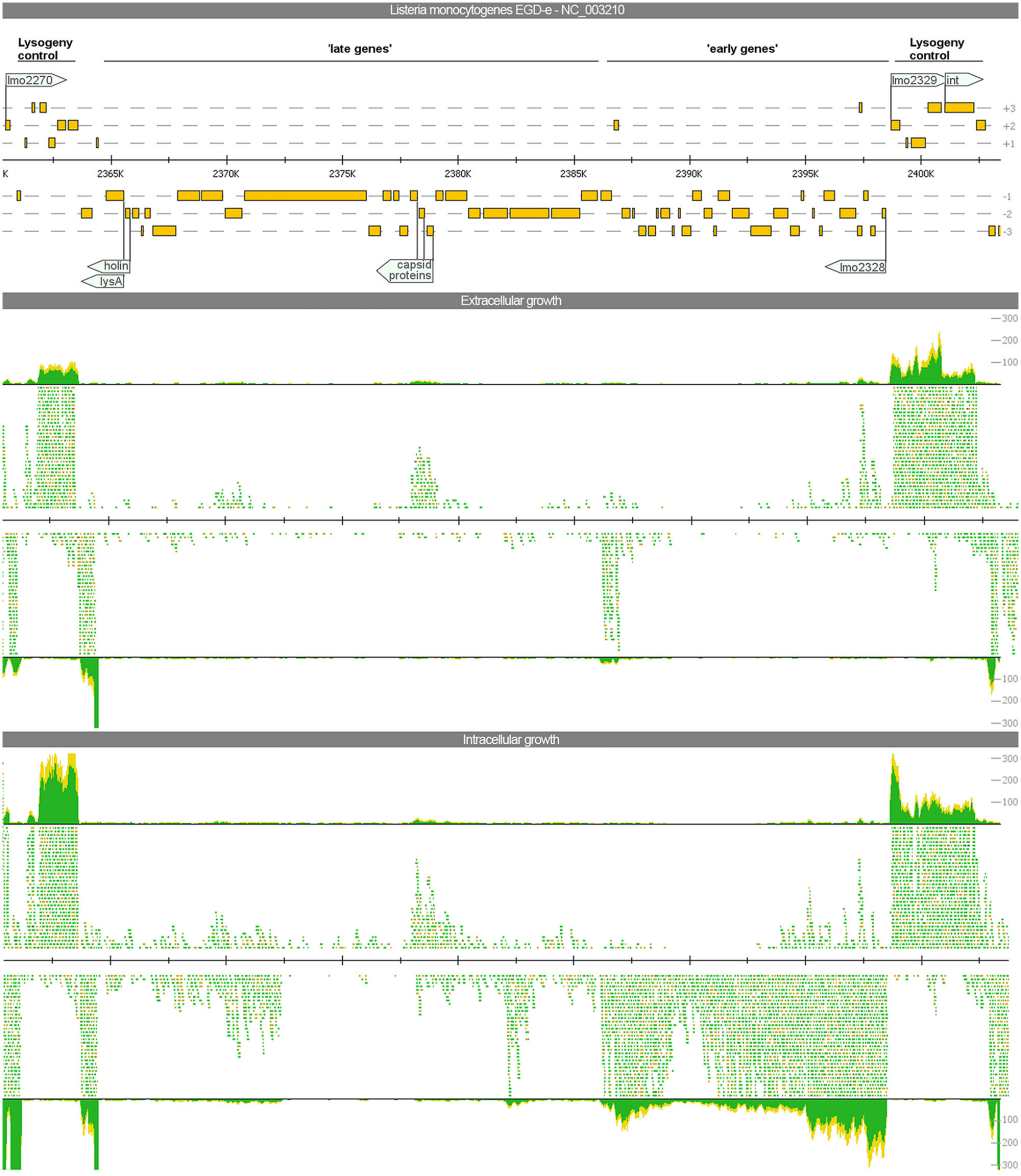
**Visualization of the gene expression profiles of the A118-like prophage locus (*lmo2271*–*2332)* under extra- and intracellular growth condition.** The read mapping illustrates a strong increase of transcripts for the ‘early genes’ whereas ‘late genes’ are poorly expressed under both conditions.

In analogy to the bacteriophage A118 described previously (Loessner et al., 2000), three organizational clusters, namely ‘late genes,’ ‘early genes,’ and ‘lysogeny control,’ can be distinguished in the phage genome. The expression among these clusters differs widely (**Figure 3**).

The late genes (*lmo2278*–*2301*) comprise genes coding for proteins required for host cell lysis, structural and assembly proteins as well as proteins for DNA packaging. All these genes are poorly expressed under any growth condition. Evidence for upregulation under intracellular growth condition was only found for *lmo2299* most likely encoding a portal protein. Beside a generally low expression, antisense transcripts were detected for all genes of this locus expressed under both investigated conditions. For the lysis factors holin and *lysA*, antisense transcripts were almost as frequent as sense transcripts and for three minor capsid proteins (*lmo2292–2294*) antisense transcripts even exceeded the amount of sense transcripts.

The early genes (*lmo2302*–*2328*), encoding for products of replication, recombination, and modification of phage DNA, showed an identical expression pattern compared to the late genes during extracellular growth. During intracellular growth, gene expression of early genes was strongly induced with 23 genes significantly upregulated.

The third cluster involved in lysogeny control (*lmo2271–2277* and *lmo2329–2332*) flanks both insertion sites and contains several putative transcriptional factors as well as the integrase. On a transcriptional level, all of these genes were well-expressed under both intra- and extracellular growth Of particular interest are the genes *lmo2328* and *lmo2329*. Both of these adjacent and divergently oriented genes possess a helix-turn-helix motif common for transcriptional regulators. This constellation is a component of several bacteriophage genomes. In *E. coli* phage aaa, homologs of these genes are involved in the switch between lysogeny and lytic behavior (Oppenheim et al., 2005). In our study, *lmo2329* is highly expressed under both conditions, but *lmo2328* is exclusively expressed under intracellular growth conditions.

**FIGURE 4:**
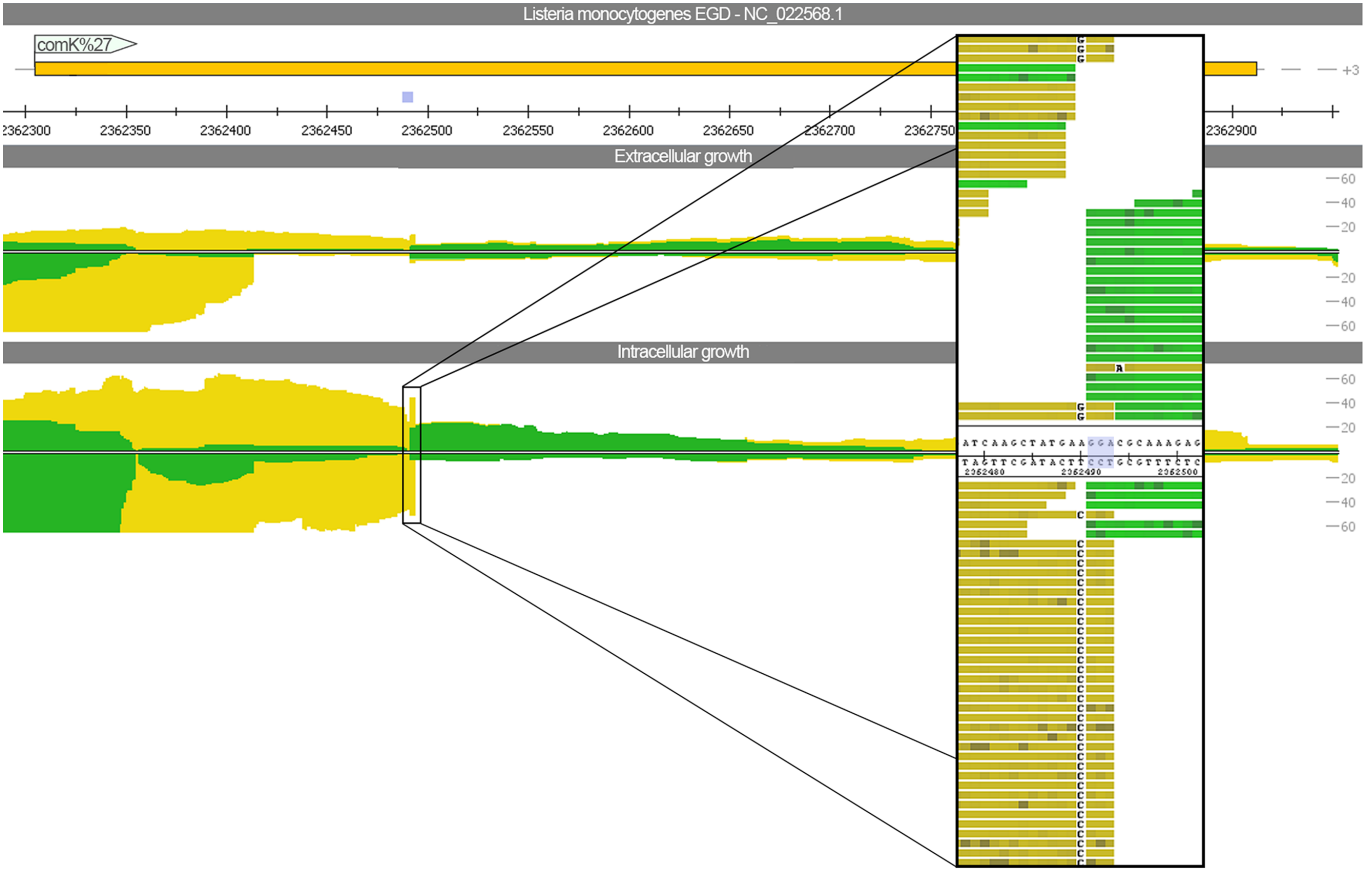
**Read mapping for intra- and extracellular growth against *Listeria monocytogenes* EGD.** In contrast to *L. monocytogenes* EGD-e, the strain EGD has no A118-like prophage insertion. The view depicts the *comK* gene, which is split and thereby inactivated by the bacteriophage insertion for the strain EGD-e. A close-up picture highlights that no reads are spanning the three nucleotide insertion site.

To assess whether phage excision takes place thereby restoring functionality of the previously split competence activator *comK* (*lmo2270* and *lmo2333*), we mapped the sequencing reads against *L. monocytogenes* EGD (downloaded from NCBI NC_022568) using relaxed mapping conditions. *L. monocytogenes* EGD is highly related to *L. monocytogenes* EGD-e but does not have the A118-like prophage insertion. The read mapping (**Figure 4**) highlights that not even a single read is spanning the phage insertion site under intracellular growth conditions. Therefore, no evidence for excision restoring *comK* was detected. In addition, no significant difference was found between intra- and extracellular growth for the gene expression of competence cluster *comF* and *comG* as well as the genes *comEA* and *comEB*. Only expression of *comEC* was increased under intracellular conditions (**Supplementary Figure S2**).

Within the phage region, *lmo2316* encoding a putative site-specific adenine methylase (based on KEGG database prediction) was highly upregulated during intracellular growth. Methylases are known to contribute to pathogenicity and intracellular survival. *lmo2316* was further characterized and the bacterial methyltransferase *lmo1119*, which is located elsewhere in the bacterial genome and is predicted to have similar functions, was included in the analysis as phage-independent control. Of note, the gene locus of *lmo1119* indicates significant difference in its GC content compared to the whole genome sequence (**Supplementary Figure S3**).

While expression of the phage gene *lmo2316* was strongly induced during intracellular growth, no difference in *lmo1119* expression was detected under intracellular growth conditions. However, *lmo1119* was well-expressed under both growth conditions whereas *lmo2316* is hardly expressed at all under extracellular growth conditions.

#### Experimental Validation of lmo2316 and lmo1119 *In vitro* and *In vivo*

**FIGURE 5:**
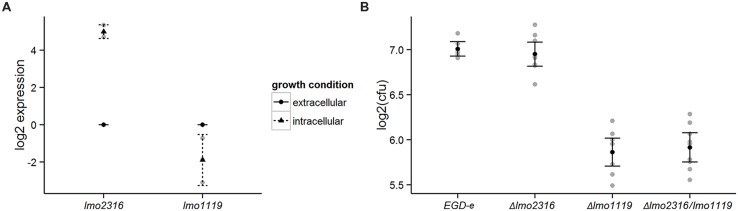
**(A)** Validation of RNA-seq data with qRT-PCR for the genes *lmo1119* and *lmo2316.* Transcripts of each gene were assessed during extracellular growth and compared to intracellular expression. Values represent means of at least three independent experiments ± 2 SE. **(B)** Survival of Δ*lmo1119*, Δ*lmo2316* and the double mutant Δ*lmo1119/lmo2316* in P388D1 murine macrophages. Macrophages were infected with wild type *L. monocytogenes* and its isogenic deletion mutants and bacterial CFU counts were measured after 4 h post-infection. Linear models to estimate intracellular survival of infection, log2-ratios of individual experiments are shown in gray. Supporting statistical information is given in Supplementary Table S4 or for a repetition of this experiment including a *prfA* deletion mutant strain as negative control in Supplementary Table S5, respectively.

We used qRT-PCR analysis to confirm the transcriptional changes of *lmo1119* and *lmo2316* indicated by RNA-seq. As predicted, *lmo2316* was significantly upregulated (*p* = 0.0014) and *lmo1119* showed a trend toward downregulation during intracellular growth (*p* = 0.1091; **Figure 5A**).

In order to assess the effect of the phage methylase on intracellular growth properties of *L. monocytogenes*, we generated a knockout mutant and investigated its ability to survive in P388D1 murine macrophages compared with the wild type strain (*L. monocytogenes* EGD-e). *lmo1119* single and *lmo1119/lmo2316* double mutants were included as controls in this experiment. Δ*lmo1119* and Δ*lmo1119/lmo2316* were significantly impaired in their intracellular survival compared to *L. monocytogenes* EGD-e. In contrast, Δ*lmo2316* survived similar to *L. monocytogenes* EGD-e. Similarly, no additive effect of Δ*lmo1119/lmo2316* was observed in comparison to Δ*lmo1119* single mutant (**Figure 5B**, **Supplementary Table S4**). Growth in BHI was not altered in the knockout strains in comparison to wild type *L. monocytogenes* EGD-e (data not shown).

In conclusion, even though *lmo2316* was significantly induced during intracellular growth, no contribution to intracellular survival of *L. monocytogenes* grown in murine macrophages was found.

## Discussion

This study reveals significant differences in the transcriptional response of *L. monocytogenes* for 23% of the genes when grown in murine macrophages compared to growth in BHI. This exceeds the microarray findings where 17% were differentially regulated (Chatterjee et al., 2006). Given the enormous differences between growth in rich media such as BHI and the cytosolic growth conditions including induction of stress responses, 23% seems reasonable if not rather low. The direct comparison between results of both studies show a high degree of similarity particularly regarding methodological differences.

This work reveals several transcriptional adaptations to intracellular growth conditions that appear to be universal, as they have been reported in previous studies after infection of epithelial cells, macrophages, and splenocytes (Chatterjee et al., 2006; Joseph et al., 2006; Camejo et al., 2009). Transcriptional upregulation of *prfA* and PrfA-dependent genes is one of those strategies. The majority of these upregulated genes are organized in the virulence gene cluster (*lmo0201–0205*). The phospholipases *plcA*, *plcB*, and listeriolysin are crucial for phagosomal escape. The metalloprotease Mpl is involved in maturation of *plcB* and the product of *actA* facilitates actin assembly beneficial for cell-to-cell spread of *Listeria* (Cossart, 2011). Apart from this locus, two internalin genes*, prsA2 and hpt* were upregulated. While Internalin B (*inlB*) is required for cell entry (Gaillard et al., 1991), internalin C (*inlC*) is involved in cell-to-cell (Engelbrecht et al., 1996; Rajabian et al., 2009) spread and interferes with the innate immune response of the host (Gouin et al., 2010). The chaperonin *lmo2219* (*prsA2*) is thought to contribute to virulence, e.g., by promoting the stability of listeriolysin (Alonzo et al., 2009; Cahoon and Freitag, 2015). Hpt is involved in the transport of sugar-phosphates and is required for efficient intracellular proliferation (Chico-Calero et al., 2002). Thus, a predominant carbon source other than glucose has been suggested during intracellular growth (Joseph et al., 2006). Our sequencing results show differential regulation of several operons involved in sugar transport. Among those, the mannose/glucose transport operon (*lmo0096–0098*) was downregulated. Expression of this operon has recently been demonstrated to play a role in glucose mediated virulence gene repression (Zébré et al., 2015).

Of note, *lmo1538*, encoding a glycerol kinase, was found to be upregulated after infection of epithelial cells, macrophages and splenocytes (Chatterjee et al., 2006; Joseph et al., 2006; Camejo et al., 2009). Therefore, glycerol represents an alternative carbon source (Joseph et al., 2008; Grubmüller et al., 2014). In this context, the downregulation of central genes for glycolysis was discussed. While this downregulation occurred for *L. monocytogenes* infecting epithelial cells and macrophages (Chatterjee et al., 2006; Joseph et al., 2006; Grubmüller et al., 2014), *L. monocytogenes* grown in spleens showed an opposite trend (Camejo et al., 2009). Our study confirmed this downregulation of several glycolysis genes and showed upregulation of an operon including a predicted glyoxolase (*lmo2671*). Thus, it is interesting to figure out whether the glyoxolase pathway, also known to function as an offshoot to glycolysis, is involved in the adaptation to intracellular growth conditions.

Another universal transcriptional response is the upregulation of genes involved in stress response. Maintenance of proper protein folding during a stress response is achieved by chaperons and ATP-dependent proteases that prevent the accumulation of misfolded proteins. Our results showed upregulation of the *dnaK*-operon and caseinolytic protease genes (*clpB*, *clpE*, and *clpC*). The importance for intracellular survival of all these caseinolytic proteases has been demonstrated (Rouquette et al., 1996; Nair et al., 1999; Chastanet et al., 2004).

In our study, we address both intracellular sense and antisense regulation in *L. monocytogenes* for the first time. We provide evidence for regulation of several genes under intracellular growth conditions. Antisense regulation in *L. monocytogenes* has not been investigated thoroughly although it might be of utmost importance for translation (Mellin and Cossart, 2012; Behrens et al., 2014; Schultze et al., 2014; Wehner et al., 2014). We found antisense regulation targeting *lmo1705*, a deoxyguanosine kinase/deoxyadenosine kinase. Read mappings of this region prompt a linkage to the transcription of the adjacent divergent operon *lmo1702–1704*. Therefore, this locus might represent a putative excludon activated under intracellular growth conditions.

In addition to the bacterial transcriptional profile, we also provide the transcriptional response of a bacteriophage to intracellular growth conditions. During the last few years, bacteriophages and their response to environmental changes were increasingly investigated as they represent a mean to counteract food-borne pathogens (Denes and Wiedmann, 2014).

The greatest transcriptional changes in the prophage region occurred in the ‘early genes.’ The function of ‘early genes’ includes DNA replication, modification, recombination, and gene expression modification. Among the upregulated genes, we focused on a site-specific DNA methylase *lmo2316*. It is well-known that DNA methylation is crucial for epigenetic control of gene expression in bacteria. Phenomena such as timing of DNA replication, DNA repair, protection against foreign bacteriophage DNA, timing of transposition, pilus expression and conjugal plasmid transfer are sensitive to the methylation states of specific DNA regions (Sánchez-Romero et al., 2015). DNA methylases are also involved in virulence (Heusipp et al., 2007). Yet, intracellular survival in macrophages was not affected by the deletion of *lmo2316*. Recent studies outline the importance of methylases in the evolutionary arms race between bacteria and bacteriophages (Labrie et al., 2010; Goldfarb et al., 2015). Given that, it is more likely that this particular DNA methylase modifies newly synthesized phage DNA to evade bacterial restriction modification (R–M) systems. Several R–M systems have been previously reported for *L. monocytogenes* epidemic clone I and II (Sau3AI-like, LmoH7, LmoJ2, and LmoJ3) which were involved in temperature dependent phage resistance (Yildirim et al., 2010; Kim et al., 2012; Lee et al., 2012). Impaired growth of Δ*lmo1119* in macrophages might be explained by the role of *lmo1119* in maintenance of the bacteria-specific DNA methylation pattern. Thus, a knockout of this gene can be detrimental due to its own R-M system. Of note, both gene loci (*lmo2316* and *lmo1119*) indicate an unusually low GC content. *lmo2316* is located in the well-known A118-like prophage locus whereas *lmo1119* is a downstream neighbor of TN916. The latter chromosomal locus was previously designated as “hypervariable hotspot 8” where LIPI-3 is inserted in lineage I strains *L. monocytogenes* (Glaser et al., 2001; Cotter et al., 2008; Kuenne et al., 2013). This suggests that both gene regions originated by horizontal gene transfer via transduction and/or transposition.

Whether the temperate phage remains in lysogenic state or switches into the lytic cycle and thereby eventually lyses its host, is of crucial importance for survival of the bacteria. For the *E. coli* phage aaa, the two adjacent and divergently oriented transcriptional regulators *cI* and *cro* are crucial for the ‘lysis-lysogeny decision.’ Gene products of *cI* repress the ‘early phage’ genes including *cro.* Gene products of *cro* in turn repress transcription of *cI* directly as well as indirectly by decreasing transcription of an activator of *cI*. Currently, the indirect route is thought to have the major effect (Dodd et al., 2005; Oppenheim et al., 2005).

Homologs of *cI* and *cro* have been described for *Lactobacillus* phage (Kodaira et al., 1997) or the *L. monocytogenes* A118 phage (Loessner et al., 2000). Similar to A118 phage, the A118-like bacteriophage in *L. monocytogenes* EGD-e contains the genes *lmo2328* and *lmo2329* exhibiting similarity to *cI* and *cro*, respectively.

However, RNA-seq data contradicts the proposed mechanism of mutual repression of *lmo2328* and *lmo2329* for the A118-like prophage. During intracellular growth, the homologs to both *cro* and *cI* were highly expressed. Hence, the mechanism of switching between lysogeny and lytic state known from *E. coli* phage aaa is probably not completely applicable in *L. monocytogenes* EGD-e.

Yet, upregulation of *lmo1398* (*recA*) during intracellular growth might explain the expression of the ‘early genes’ including *lmo2328* (homolog to *cro*) despite the presence of *lmo2329* (homolog to the inhibitor of early genes *cI*) transcripts. In *E. coli*, RecA supports cleavage of *cI* and thereby derepresses the ‘early genes.’

Of note, the ‘late genes’ are only poorly expressed under both growth conditions. The discovery of antisense transcripts in this region indicates another inhibitory mechanism.

Our study also provided further insights concerning the excision of the A118-like prophage of *L. monocytogenes* EGD-e when grown in macrophages. In *L. monocytogenes* strain 10403S a comparable phage insertion was investigated in murine bone-marrow derived macrophages under intracellular growth conditions (Rabinovich et al., 2012). This study demonstrated that prophage excision takes place under intracellular growth conditions thereby restoring functionality of the previously split competence activator *comK* (Rabinovich et al., 2012). We found no evidence for such an excision of the prophage genome. This is corroborated by unaltered transcript expression of the phage integrase under both growth conditions.

Moreover, the increased gene expression of all three competence gene clusters (*comE*, *comG*, and *comK*) in *L. monocytogenes* 10403S due to restoration of *comK* (Rabinovich et al., 2012) was not confirmed for *L. monocytogenes* EGD-e. In contrast, our transcriptional profile showed no intracellular upregulation of the clusters *comG* and *comK* as well as no changes in *comEA* and *comEB* expression. Only *comEC* expression was intracellularly upregulated in our study. Interestingly, only *comG* and *comEC* were shown to be beneficial for intracellular survival probably by alleviating the phagolysosomal escape (Rabinovich et al., 2012). We propose that in *L. monocytogenes* EGD-e, *comEC* can be alternatively activated bypassing the global competence activator *comK*.

## Conclusion

Here, we present the first intracellular mRNA transcriptome for *L. monocytogenes* obtained using an NGS approach. RNA-seq-based studies have the power to study both coding and non-coding RNA phenomena. Our data is supported by previous microarray studies. In summary, we provide a base for future work targeting intracellular survival of *L. monocytogenes* covering both sense and antisense transcript changes. By comparing mRNA transcriptome data from different studies and different stages of listerial infection (epithelial cells, macrophages, and spleen as target organ), we identified a common set of genes always upregulated during intracellular growth. Furthermore, we provide the transcriptional profile of a temperate A118-like phage thereby offering novel insights in phage control and the intertwined coexisting between bacteriophage and bacteria. Taken together, this work expands the view on the transcriptional changes arising from intracellular growth conditions.

## Conflict of Interest Statement

The authors declare that the research was conducted in the absence of any commercial or financial relationships that could be construed as a potential conflict of interest.

## Footnotes

1 http://CRAN.R-project.org/package=lsmeans

2 http://www.ncbi.nlm.nih.gov/COG/

## Supplementary Material

**FIGURE S1 |Coverage plot of all operons for which differential expression patterns were in discord with the predicted operon model** (Toledo-Arana et al., 2009). SigB operon (*lmo0887-0896*) shown in **(A)**, *lmo1416-1420* in **(B)** and *lmo2463-2465* given in **(C)**.

**FIGURE S2 |Read mappings of all competence clusters.** The compentence cluster comG and comF are poorly expressed. Only *comEC* (*lmo1482*) was significantly upregulated during intracellular growth.

**FIGURE S3 |Visualization of the chromosomal regions of *lmo1119***(A)** and *lmo2316***(B)** using GECO** (Kuenne et al., 2007). The line below the illustrated genes indicates the deviation from the average GC content. Values greater than zero are highlighted by brown colour while those below zero are blue. **(C)** Comparison of the A118-like prophage region between the *Listeria monocytogenes* strains EGD-e, 10403S and EGD. Genes are colored according to similarity on protein level. Core genes are indicated in black.
