# Supplementary figures and images for: A detailed view of the intracellular transcriptome of Listeria monocytogenes in murine macrophages using RNA-seq
Source: Front Microbiol. 2015 Oct 30;6:1199. doi: 10.3389/fmicb.2015.01199 (PMC4627465; doi:10.3389/fmicb.2015.01199)

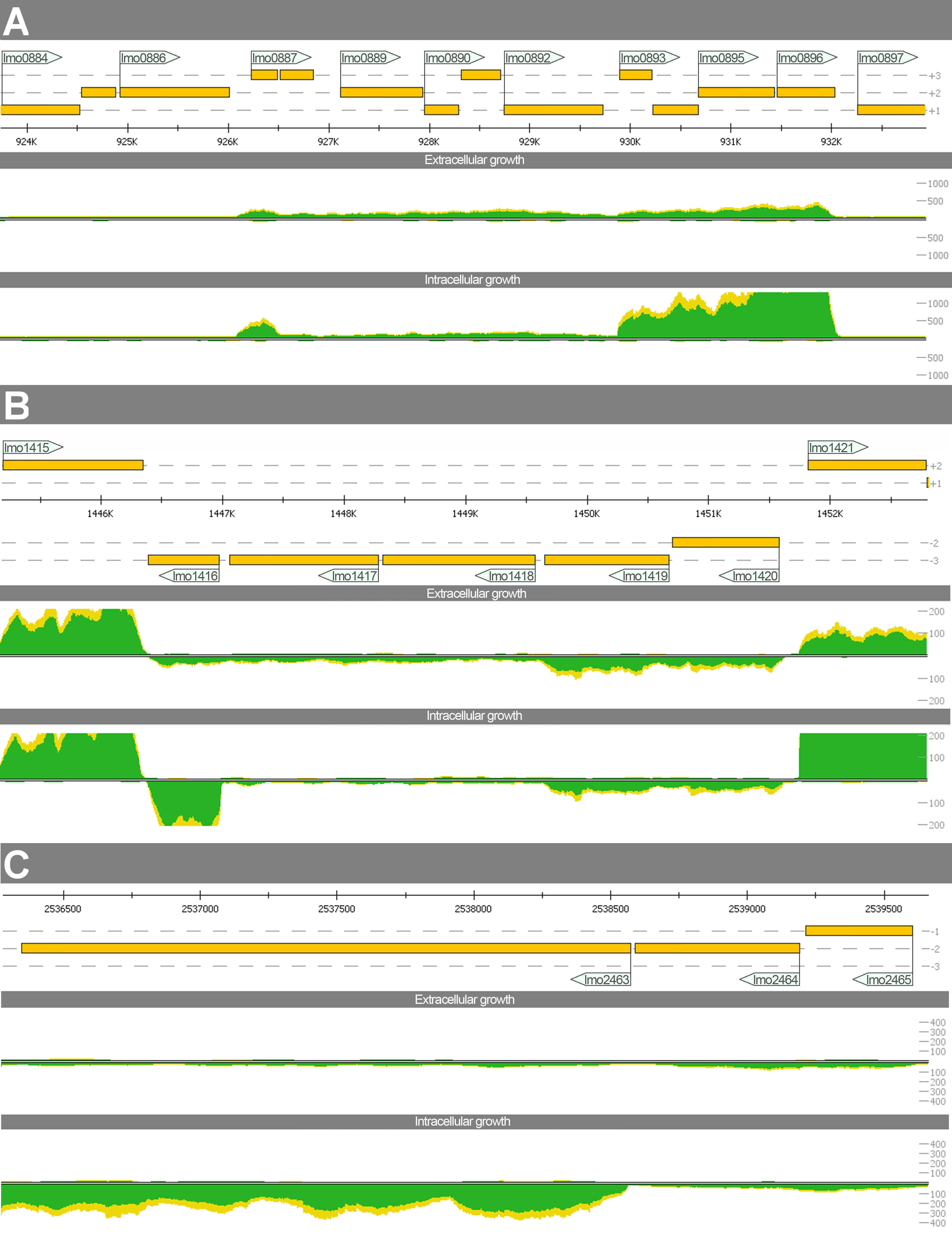

Supplement: Supplementary file 1 [file Supplementary_Figure_S1.TIF]

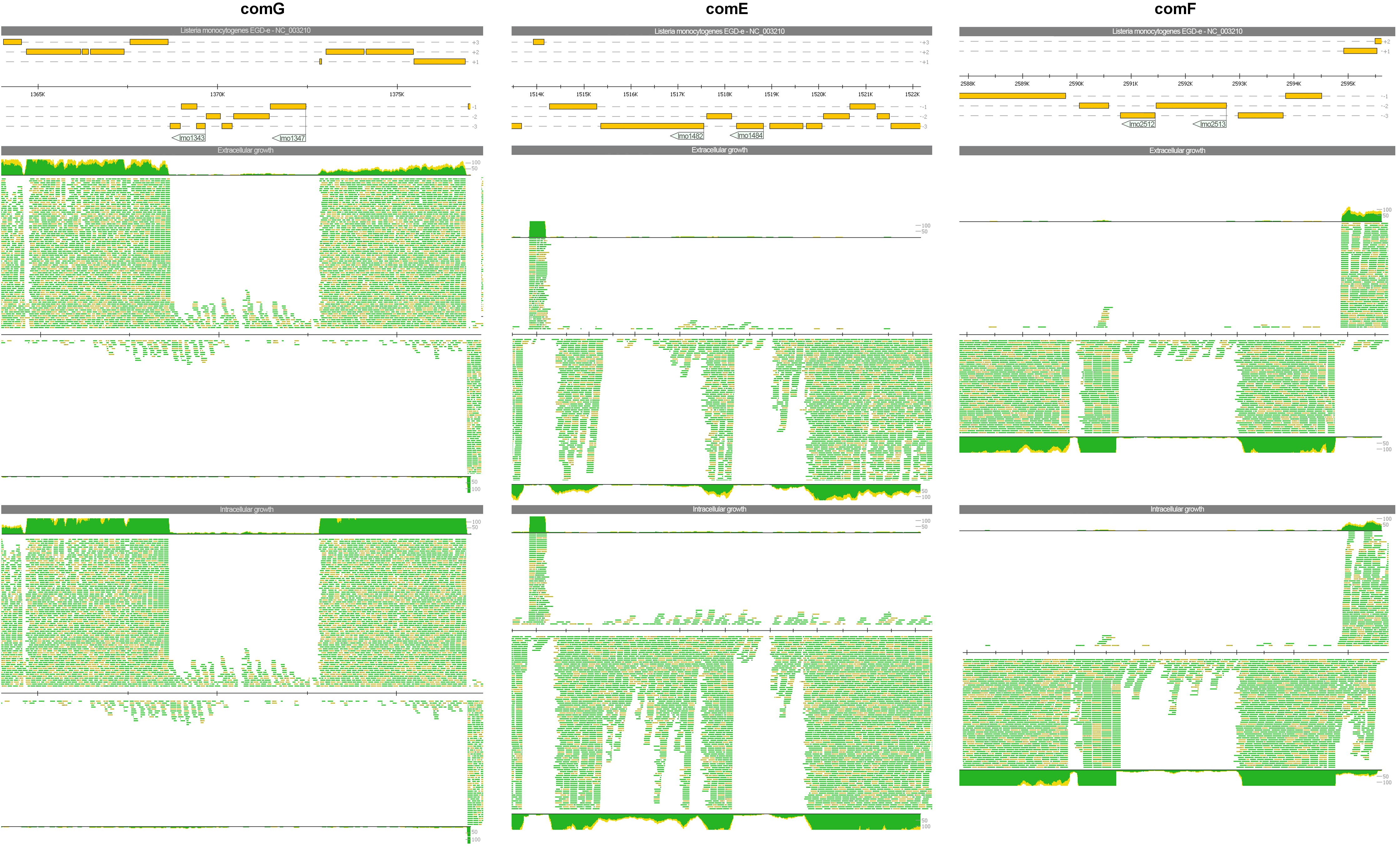

Supplement: Supplementary file 2 [file Supplementary_Figure_S2.TIF]

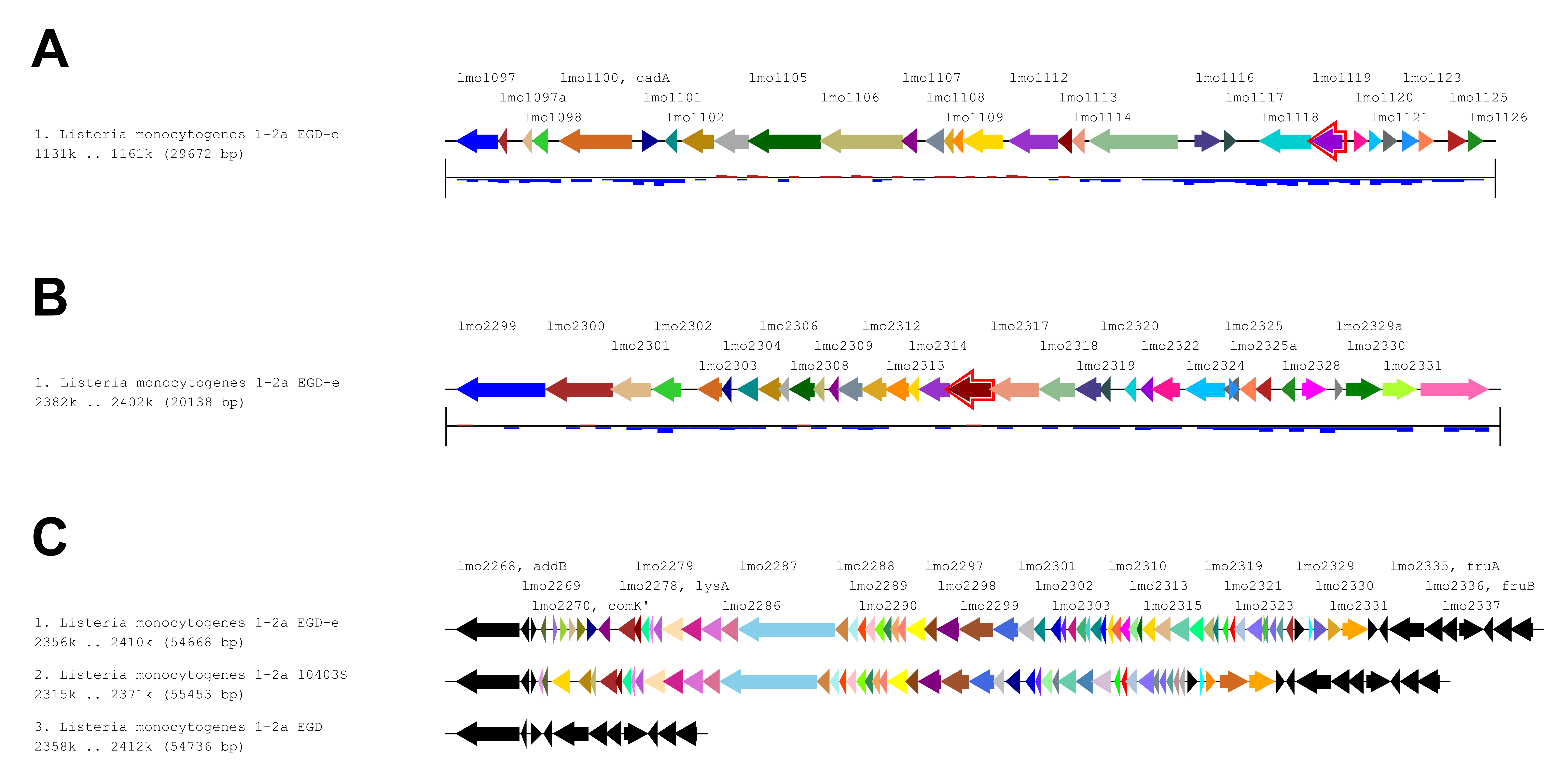

Supplement: Supplementary file 3 [file Supplementary_Figure_S3.TIF]
