# Supplementary material for: A detailed view of the intracellular transcriptome of Listeria monocytogenes in murine macrophages using RNA-seq
Source: Front Microbiol. 2015 Oct 30;6:1199. doi: 10.3389/fmicb.2015.01199 (PMC4627465; doi:10.3389/fmicb.2015.01199)
Supplement: Supplementary file 4 [file Supplementary_Tables.DOCX]

**Supplemental material**

**Table S1** Primers used in this study.

| **Name** | **5´-3´ Sequence** |
| --- | --- |
| lmo1119_1 | ATTAACGTCCATATAGTCAG |
| lmo1119_2 | GCGCTAAATCTGGGTTCATTTTGTTTCCTCC |
| lmo1119_3 | AATGAACCCAGATTTAGCGCCAGAGATTAGC |
| lmo1119_4 | TAGACTTGCATTTACCAAACC |
| lmo1119_7 | TTAGTACATTTTGAGTATCGG |
| lmo1119_8 | ATCTTCTCCTGAGTGTATACG |
| lmo2316_1 | ATAGCTGATGTTGATGCAGCGG |
| lmo2316_2 | CTGATAACTCTGCACATGATCCGCAGAATGG |
| lmo2316_3 | ATCATGTGCATTATCAGACTGGCCAGTGC |
| lmo2316_4 | GGCAAACCGAGGAACGGCTA |
| lmo2316_7 | AAACATCGAGAAGCTATGCGG |
| lmo2316_8 | TCGTTCCATGTTGAAAGCGGC |
| lmo1119_5’ | TATCCTGGTGGTAAACATA |
| lmo1119_3’ | ATCAAGGACACTTTTCCAAA |
| lmo2316_5’ | TACACTGATATATTGTGACCCG |
| lmo2316_3’ | TCTGATTCGTAACCGCTTAAC |

**Table S2** Differentially expressed genes when grown in murine macrophages compared to BHI.

| **Locus tag** | **Gene** | **Description** | **log2FC** |
| --- | --- | --- | --- |
| *lmo0205* | *plcB* | phospholipase C | 7.56 |
| *lmo0203* | *mpl* | Zinc metalloproteinase precursor | 7.28 |
| *lmo2751* | *lmo2751* | ABC transporter ATP-binding protein | 5.93 |
| *lmo0204* | *actA* | actin-assembly inducing protein precursor | 6.94 |
| *lmo1786* | *inlC* | internalin C | 6.85 |
| *lmo0202* | *hly* | listeriolysin O precursor | 5.15 |
| *lmo0207* | *lmo0207* | hypothetical protein | 5.81 |
| *lmo0838* | *uhpT* | sugar:phosphate antiporter | 6.29 |
| *lmo0608* | *lmo0608* | ABC transporter ATP-binding protein | 4.80 |
| *lmo2828* | *lmo2828* | hypothetical protein | 5.69 |
| *lmo0206* | *lmo0206* | hypothetical protein | 7.00 |
| *lmo0751* | *lmo0751* | hypothetical protein | 6.48 |
| *lmo2752* | *lmo2752* | ABC transporter ATP-binding protein | 5.02 |
| *lmo0576* | *lmo0576* | hypothetical protein | 4.93 |
| *lmo0955* | *lmo0955* | hypothetical protein | 3.80 |
| *lmo0514* | *lmo0514* | internalin | 4.26 |
| *lmo0749* | *lmo0749* | hypothetical protein | 7.05 |
| *lmo2102* | *lmo2102* | glutamine amidotransferase subunit PdxT | -5.37 |
| *lmo0954* | *lmo0954* | hypothetical protein | 4.32 |
| *lmo0748* | *lmo0748* | hypothetical protein | 6.28 |
| *lmo0750* | *lmo0750* | hypothetical protein | 6.18 |
| *lmo2827* | *lmo2827* | MarR family transcriptional regulator | 5.19 |
| *lmo0607* | *lmo0607* | ABC transporter ATP-binding protein | 3.83 |
| *lmo0201* | *plcA* | phosphatidylinositol-specific phospholipase c | 5.64 |
| *lmo0496* | *lmo0496* | hypothetical protein | 4.73 |
| *lmo0991* | *lmo0991* | hypothetical protein | 4.15 |
| *lmo2101* | *lmo2101* | pyridoxal biosynthesis lyase PdxS | -4.38 |
| *lmo2224* | *lmo2224* | hypothetical protein | 3.64 |
| *lmo1652* | *lmo1652* | ABC transporter ATP-binding protein | 3.42 |
| *lmo1289* | *lmo1289* | internalin | 3.82 |
| *lmo2826* | *lmo2826* | MFS transporter | 4.67 |
| *lmo2323* | *lmo2323* | hypothetical protein gp43 [Bacteriophage A118] | 6.15 |
| *lmo2328* | *lmo2328* | XRE family transcriptional regulator | 6.46 |
| *lmo1974* | *lmo1974* | GntR family transcriptional regulator | 4.70 |
| *lmo2317* | *lmo2317* | hypothetical protein | 6.29 |
| *lmo1651* | *lmo1651* | ABC transporter ATP-binding protein | 3.50 |
| *lmo1137* | *lmo1137* | hypothetical protein | 4.77 |
| *lmo2324* | *lmo2324* | anti-repressor | 5.88 |
| *lmo2327* | *lmo2327* | hypothetical protein | 6.71 |
| *lmo1870* | *lmo1870* | alkaline phosphatase | 3.69 |
| *lmo2318* | *lmo2318* | hypothetical protein | 5.78 |
| *lmo2386* | *lmo2386* | hypothetical protein | 3.10 |
| *lmo1970* | *lmo1970* | phosphotriesterase | 5.88 |
| *lmo0752* | *lmo0752* | hypothetical protein | 5.10 |
| *lmo2316* | *lmo2316* | site-specific DNA-methyltransferase | 5.25 |
| *lmo0857* | *lmo0857* | carboxylesterase | -2.71 |
| *lmo2779* | *ychF* | GTP-binding protein EngD | -2.55 |
| *lmo1690* | *lmo1690* | hypothetical protein | 3.20 |
| *lmo0520* | *lmo0520* | transcriptional regulator | 4.80 |
| *lmo1975* | *lmo1975* | DNA polymerase IV | 3.45 |
| *lmo2303* | *lmo2303* | hypothetical protein | 4.77 |
| *lmo2672* | *lmo2672* | AraC family transcriptional regulator | 3.54 |
| *lmo2733* | *lmo2733* | PTS fructose transporter subunit IIABC | 3.46 |
| *lmo1973* | *lmo1973* | PTS sugar transporter subunit IIA | 6.21 |
| *lmo2829* | *lmo2829* | nitroreductase | 3.35 |
| *lmo0200* | *prfA* | listeriolysin positive regulatory protein | 3.77 |
| *lmo2494* | *lmo2494* | PhoU family transcriptional regulator | 3.88 |
| *lmo2671* | *lmo2671* | hypothetical protein | 3.07 |
| *lmo1425* | *opuCD* | glycine/betaine ABC transporter permease | 3.01 |
| *lmo1971* | *ulaA* | PTS ascorbate transporter subunit IIC membrane component | 5.39 |
| *lmo0818* | *lmo0818* | cation-transporting ATPase | 3.14 |
| *lmo2460* | *lmo2460* | transcriptional regulator | -3.06 |
| *lmo2258* | *lmo2258* | hypothetical protein | 3.56 |
| *lmo2322* | *lmo2322* | gp44 gp44 [Bacteriophage A118] | 6.36 |
| *lmo0047* | *lmo0047* | hypothetical protein | 2.80 |
| *lmo0753* | *lmo0753* | Crp/Fnr family transcriptional regulator | 4.06 |
| *lmo2326* | *lmo2326* | hypothetical protein | 5.71 |
| *lmo1389* | *lmo1389* | sugar ABC transporter ATP-binding protein | -2.58 |
| *lmo0232* | *clpC* | endopeptidase Clp ATP-binding chain C | 2.96 |
| *lmo1806* | *acpP* | acyl carrier protein | -2.47 |
| *lmo0997* | *clpE* | ATP-dependent protease | 4.99 |
| *lmo2487* | *lmo2487* | hypothetical protein | 4.40 |
| *lmo0604* | *lmo0604* | hypothetical protein | 4.61 |
| *lmo1997* | *lmo1997* | PTS mannose transporter subunit IIA | 4.17 |
| *lmo2230* | *lmo2230* | arsenate reductase | 4.10 |
| *lmo2766* | *lmo2766* | RpiR family transcriptional regulator | -3.02 |
| *lmo2321* | *lmo2321* | hypothetical protein | 5.75 |
| *lmo0937* | *lmo0937* | hypothetical protein | 3.11 |
| *lmo2206* | *clpB* | Clp protease subunit B | 3.81 |
| *lmo1968* | *lmo1968* | creatinine amidohydrolase | 4.49 |
| *lmo1252* | *lmo1252* | hypothetical protein | 2.39 |
| *lmo1472* | *dnaJ* | molecular chaperone DnaJ chaperone Hsp40 | 3.16 |
| *lmo0754* | *lmo0754* | hypothetical protein | 3.81 |
| *lmo1650* | *lmo1650* | hypothetical protein | 2.55 |
| *lmo2325* | *lmo2325* | hypothetical protein | 6.09 |
| *lmo2485* | *lmo2485* | hypothetical protein | 3.47 |
| *lmo2313* | *lmo2313* | hypothetical protein | 5.46 |
| *lmo2484* | *lmo2484* | hypothetical protein | 3.64 |
| *lmo0048* | *lmo0048* | sensor histidine kinase AgrB essential for production of a quorum sensing autoinducing peptide | -2.93 |
| *lmo1603* | *lmo1603* | aminopeptidase | -3.03 |
| *lmo2315* | *lmo2315* | hypothetical protein | 4.93 |
| *lmo0051* | *lmo0051* | response regulator | -2.91 |
| *lmo1998* | *lmo1998* | opine catabolism protein | 4.40 |
| *lmo2497* | *lmo2497* | phosphate ABC transporter permease | 3.01 |
| *lmo2489* | *uvrB* | excinuclease ABC subunit B | 2.84 |
| *lmo0591* | *lmo0591* | hypothetical protein | 2.47 |
| *lmo1388* | *tcsA* | CD4+ T cell-stimulating antigen, lipoprotein | -2.92 |
| *lmo2856* | *rpmH* | 50S ribosomal protein L34 | -1.94 |
| *lmo0849* | *lmo0849* | amidase EC_Number 3.5.1.4 | 3.15 |
| *lmo0372* | *lmo0372* | beta-glucosidase | 4.07 |
| *lmo0254* | *lmo0254* | hypothetical protein | 3.88 |
| *lmo0980* | *lmo0980* | ABC transporter permease | 3.22 |
| *lmo2439* | *lmo2439* | hypothetical protein | 3.74 |
| *lmo2308* | *lmo2308* | single-stranded DNA-binding protein | 4.46 |
| *lmo2271* | *lmo2271* | hypothetical protein | 3.38 |
| *lmo0871* | *lmo0871* | hypothetical protein | 3.21 |
| *lmo2498* | *lmo2498* | phosphate ABC transporter permease | 4.34 |
| *lmo1105* | *lmo1105* | hypothetical protein | 3.37 |
| *lmo0956* | *lmo0956* | N-acetylglucosamine-6P-phosphate deacetylase | 2.76 |
| *lmo1999* | *lmo1999* | hypothetical protein | 4.54 |
| *lmo0257* | *lmo0257* | hypothetical protein | 2.65 |
| *lmo0410* | *lmo0410* | pseudo | 2.45 |
| *lmo0910* | *lmo0910* | hypothetical protein | 2.57 |
| *lmo2676* | *lmo2676* | DNA polymerase IV | 2.88 |
| *lmo1966* | *lmo1966* | hypothetical protein | 3.16 |
| *lmo2345* | *lmo2345* | hypothetical protein | -4.09 |
| *lmo2248* | *lmo2248* | hypothetical protein | -2.38 |
| *lmo2495* | *lmo2495* | phosphate ABC transporter ATP-binding protein ATP-binding protein | 3.65 |
| *lmo1081* | *lmo1081* | glucose-1-phosphate thymidyl transferase | -2.08 |
| *lmo1848* | *lmo1848* | metal ABC transporter permease | 2.56 |
| *lmo2319* | *lmo2319* | hypothetical protein | 4.65 |
| *lmo2000* | *lmo2000* | PTS mannose transporter subunit IID | 4.95 |
| *lmo2050* | *lmo2050* | excinuclease ABC subunit A | 2.50 |
| *lmo2567* | *lmo2567* | hypothetical protein | 4.40 |
| *lmo2735* | *lmo2735* | sucrose phosphorylase | 3.05 |
| *lmo0670* | *lmo0670* | hypothetical protein | 2.64 |
| *lmo2249* | *lmo2249* | low-affinity inorganic phosphate transporter | -2.37 |
| *lmo0373* | *lmo0373* | PTS beta-glucoside transporter subunit IIC | 3.73 |
| *lmo0647* | *lmo0647* | hypothetical protein | 2.53 |
| *lmo2488* | *uvrA* | excinuclease ABC subunit A | 2.45 |
| *lmo1788* | *lmo1788* | transcriptional regulator | 2.90 |
| *lmo1138* | *lmo1138* | ATP-dependent Clp protease proteolytic subunit | 2.92 |
| *lmo1294* | *miaA* | tRNA delta(2)-isopentenylpyrophosphate transferase IPP transferase | -1.94 |
| *lmo1418* | *lmo1418* | hypothetical protein | -3.06 |
| *lmo2343* | *lmo2343* | nitrilotriacetate monooxygenase | -3.11 |
| *lmo1967* | *lmo1967* | toxic ion resistance protein | 2.94 |
| *lmo0673* | *lmo0673* | hypothetical protein | 2.58 |
| *lmo2155* | *nrdA* | ribonucleotide-diphosphate reductase subunit alpha EC_Number 1.17.4.1 | -2.40 |
| *lmo1828* | *lmo1828* | hypothetical protein | -2.40 |
| *lmo2449* | *lmo2449* | exoribonuclease RNase-R | 1.88 |
| *lmo2769* | *lmo2769* | ABC transporter ATP-binding protein | -2.41 |
| *lmo2496* | *lmo2496* | phosphate ABC transporter ATP-binding protein ATP-binding protein | 2.93 |
| *lmo0820* | *lmo0820* | acetyltransferase | 2.37 |
| *lmo2690* | *lmo2690* | TetR family transcriptional regulator | 2.49 |
| *lmo1865* | *lmo1865* | hypothetical protein | 2.33 |
| *lmo1416* | *lmo1416* | hypothetical protein | 2.01 |
| *lmo2691* | *murA* | autolysin cell wall hydrolase | -2.52 |
| *lmo2716* | *cydC* | ABC transporter | -2.08 |
| *lmo0255* | *lmo0255* | hypothetical protein | 3.56 |
| *lmo0354* | *lmo0354* | fatty-acid--CoA ligase | -2.28 |
| *lmo1257* | *lmo1257* | hypothetical protein | -3.78 |
| *lmo2570* | *lmo2570* | hypothetical protein | 3.19 |
| *lmo0112* | *lmo0112* | Fnr/Crp family transcriptional regulator | 2.37 |
| *lmo1303* | *lmo1303* | cell division suppressor | 3.12 |
| *lmo2114* | *lmo2114* | ABC transporter ATP-binding protein | 2.94 |
| *lmo2164* | *lmo2164* | AraC family transcriptional regulator | -2.56 |
| *lmo2637* | *lmo2637* | hypothetical protein | -3.28 |
| *lmo2670* | *lmo2670* | hypothetical protein | 2.60 |
| *lmo0825* | *lmo0825* | 3-hydroxy-3-methylglutaryl-CoA reductase | -2.06 |
| *lmo2720* | *lmo2720* | acetate-CoA ligase | -2.68 |
| *lmo0738* | *lmo0738* | PTS beta-glucoside transporter subunit IIABC | 3.16 |
| *lmo1454* | *rpoD* | RNA polymerase sigma factor RpoD | 2.03 |
| *lmo1704* | *lmo1704* | hypothetical protein | 2.53 |
| *lmo1024* | *lmo1024* | hypothetical protein | -2.38 |
| *lmo2001* | *lmo2001* | PTS mannose transporter subunit IIC | 4.20 |
| *lmo2002* | *lmo2002* | PTS mannose transporter subunit IIB | 4.42 |
| *lmo1733* | *gltD* | glutamate synthase subunit beta EC_Number 1.4.1.13 | 2.70 |
| *lmo2257* | *lmo2257* | hypothetical protein | 2.60 |
| *lmo2675* | *lmo2675* | hypothetical protein | 2.61 |
| *lmo2736* | *lmo2736* | hypothetical protein | 3.12 |
| *lmo1962* | *lmo1962* | TetR family transcriptional regulator | 2.67 |
| *lmo1551* | *folC* | folyl-polyglutamate synthetase | -2.52 |
| *lmo0130* | *lmo0130* | 5'-nucleotidase | -2.51 |
| *lmo0855* | *ddl* | D-alanyl-alanine synthetase A EC_Number 6.3.2.4 | -1.97 |
| *lmo0231* | *lmo0231* | ATP:guanido phosphotransferase | 2.82 |
| *lmo0911* | *lmo0911* | hypothetical protein | 3.03 |
| *lmo2156* | *lmo2156* | hypothetical protein | 3.14 |
| *lmo0434* | *inlB* | internalin B | 2.76 |
| *lmo0558* | *lmo0558* | hypothetical protein | -2.07 |
| *lmo2190* | *mecA* | adaptor protein | 2.07 |
| *lmo2734* | *lmo2734* | sugar hydrolase | 2.33 |
| *lmo0590* | *lmo0590* | hypothetical protein | 2.14 |
| *lmo0096* | *lmo0096* | PTS mannose transporter subunit IIAB | -2.48 |
| *lmo2132* | *lmo2132* | hypothetical protein | 3.00 |
| *lmo0585* | *lmo0585* | secreted protein | 2.27 |
| *lmo1459* | *glyQ* | glycyl-tRNA synthetase subunit alpha EC_Number 6.1.1.14 | -2.01 |
| *lmo1952* | *lysA* | diaminopimelate decarboxylase | -1.82 |
| *lmo2064* | *mscL* | large-conductance mechanosensitive channel protein homopentamer | -2.38 |
| *lmo2745* | *lmo2745* | ABC transporter ATP-binding protein | 2.22 |
| *lmo2305* | *lmo2305* | hypothetical protein | 3.84 |
| *lmo2346* | *lmo2346* | amino acid ABC transporter ATP-binding protein | -3.52 |
| *lmo0050* | *lmo0050* | histidine kinase | -2.83 |
| *lmo0778* | *lmo0778* | hypothetical protein | -3.04 |
| *lmo2129* | *lmo2129* | hypothetical protein | -2.63 |
| *lmo0839* | *lmo0839* | tetracycline resistance protein | 2.97 |
| *lmo1498* | *lmo1498* | O-methyltransferase | -2.46 |
| *lmo0821* | *lmo0821* | hypothetical protein | 2.35 |
| *lmo2320* | *lmo2320* | hypothetical protein | 4.74 |
| *lmo2499* | *lmo2499* | phosphate ABC transporter substrate-binding protein | 3.74 |
| *lmo1400* | *lmo1400* | N-acetyltransferase | -2.01 |
| *lmo1080* | *lmo1080* | teichoic acid biosynthesis protein GgaB | -1.92 |
| *lmo2304* | *lmo2304* | hypothetical protein | 4.91 |
| *lmo2087* | *lmo2087* | hypothetical protein | 2.52 |
| *lmo1417* | *lmo1417* | hypothetical protein | -2.77 |
| *lmo1458* | *glyS* | glycyl-tRNA synthetase subunit beta glycine--tRNA ligase beta chain | -1.96 |
| *lmo1725* | *lmo1725* | GntR family tramscriptional regulator | 2.32 |
| *lmo0896* | *rsbX* | indirect negative regulation of sigma B dependant gene expression (serine phosphatase) | 1.72 |
| *lmo0646* | *lmo0646* | hypothetical protein | 2.08 |
| *lmo1810* | *lmo1810* | fatty acid biosynthesis transcriptional regulator | -2.45 |
| *lmo1446* | *zurM* | metal (zinc) transport protein (ABC transporter, permease) | 1.92 |
| *lmo2463* | *lmo2463* | multidrug transporter | 2.55 |
| *lmo1361* | *xseA* | exodeoxyribonuclease VII large subunit EC_Number 3.1.11.6 | -1.78 |
| *lmo2226* | *lmo2226* | hypothetical protein | 2.34 |
| *lmo0909* | *lmo0909* | GntR family transcriptional regulator | 2.30 |
| *lmo1231* | *lmo1231* | DNA polymerase beta | 1.78 |
| *lmo0919* | *lmo0919* | antibiotic ABC transporter ATP-binding protein | 2.06 |
| *lmo1538* | *glpK* | glycerol kinase EC_Number 2.7.1.30 | 2.09 |
| *lmo1405* | *lmo1405* | anti-terminator regulatory protein | -1.77 |
| *lmo2003* | *lmo2003* | GntR family transcriptional regulator | 3.86 |
| *lmo0541* | *lmo0541* | ABC transporter substrate-binding protein | -3.78 |
| *lmo2203* | *lmo2203* | N-acetylmuramoyl-L-alanine amidase | 2.21 |
| *lmo1427* | *opuCB* | glycine/betaine ABC transporter permease | 2.26 |
| *lmo2749* | *lmo2749* | glutamine amidotransferase | -2.46 |
| *lmo0796* | *lmo0796* | hypothetical protein | 2.62 |
| *lmo2177* | *lmo2177* | hypothetical protein | 2.69 |
| *lmo0856* | *murF* | UDP-N-acetylmuramoylalanyl-D-glutamyl-2,6-diamino pimelate-D-alanyl-D-alanyl ligase | -2.26 |
| *lmo0823* | *lmo0823* | oxidoreductase | -2.20 |
| *lmo1403* | *mutS* | DNA mismatch repair protein MutS | -1.50 |
| *lmo0484* | *lmo0484* | heme-degrading monooxygenase IsdG EC_Number 1.14.99.3 | -3.59 |
| *lmo1026* | *lmo1026* | LytR protein | 1.94 |
| *lmo2149* | *lmo2149* | hypothetical protein | -2.67 |
| *lmo1969* | *lmo1969* | 2-keto-3-deoxygluconate-6-phosphate aldolase | 3.84 |
| *lmo2088* | *lmo2088* | transcriptional regulator | 2.41 |
| *lmo0584* | *lmo0584* | hypothetical protein | 2.39 |
| *lmo1604* | *lmo1604* | 2-cys peroxiredoxin | -2.04 |
| *lmo2349* | *lmo2349* | amino acid ABC transporter substrate-binding protein | -2.99 |
| *lmo0486* | *rpmF* | 50S ribosomal protein L32 | -1.87 |
| *lmo0374* | *lmo0374* | PTS beta-glucoside transporter subunit IIB | 3.31 |
| *lmo0606* | *lmo0606* | MarR family transcriptional regulator | 2.86 |
| *lmo0077* | *lmo0077* | hypothetical protein | -2.56 |
| *lmo0736* | *lmo0736* | ribose-5-phosphate isomerase B EC_Number 5.3.1.6 | 4.66 |
| *lmo1685* | *gsaB* | glutamate-1-semialdehyde aminotransferase EC_Number 5.4.3.8 | -1.80 |
| *lmo2311* | *lmo2311* | hypothetical protein | 4.30 |
| *lmo2538* | *upp* | uracil phosphoribosyltransferase | -1.82 |
| *lmo2131* | *lmo2131* | hypothetical protein | 2.99 |
| *lmo0883* | *lmo0883* | hypothetical protein | -1.56 |
| *lmo2231* | *lmo2231* | hypothetical protein | 2.52 |
| *lmo1095* | *lmo1095* | PTS cellbiose transporter subunit IIB | -2.22 |
| *lmo1520* | *hisS* | histidyl-tRNA synthetase EC_Number 6.1.1.21 | -1.79 |
| *lmo2351* | *lmo2351* | FMN reductase | -4.00 |
| *lmo0759* | *lmo0759* | hypothetical protein | 2.18 |
| *lmo2416* | *lmo2416* | hypothetical protein | -1.63 |
| *lmo0892* | *rsbU* | serine phosphatase | -1.94 |
| *lmo0815* | *lmo0815* | transcriptional regulator | 2.38 |
| *lmo1374* | *lmo1374* | branched-chain alpha-keto acid dehydrogenase subunit E2 | -1.79 |
| *lmo1519* | *aspS* | aspartyl-tRNA synthetase EC_Number 6.1.1.12 | -1.48 |
| *lmo2210* | *lmo2210* | hypothetical protein | 2.93 |
| *lmo1424* | *lmo1424* | manganese transporter | 1.52 |
| *lmo0477* | *lmo0477* | secreted protein | -2.47 |
| *lmo2466* | *lmo2466* | hypothetical protein | -3.17 |
| *lmo0195* | *lmo0195* | ABC transporter permease | 1.75 |
| *lmo1369* | *lmo1369* | phosphotransbutyrylase | -2.76 |
| *lmo1220* | *lmo1220* | hypothetical protein | 2.09 |
| *lmo2004* | *lmo2004* | GntR family transcriptional regulator | 2.88 |
| *lmo1856* | *deoD* | purine nucleoside phosphorylase | -1.69 |
| *lmo1447* | *zurA* | metal (zinc) transport protein(ABC transporter, ATP-binding protein) | 1.76 |
| *lmo0760* | *lmo0760* | hypothetical protein | 2.31 |
| *lmo1398* | *recA* | recombinase A | 2.32 |
| *lmo0481* | *lmo0481* | hypothetical protein | 1.53 |
| *lmo2559* | *pyrG* | CTP synthetase EC_Number 6.3.4.2 | -2.25 |
| *lmo2128* | *lmo2128* | LacI family transcriptional regulator | -1.78 |
| *lmo1409* | *lmo1409* | multidrug transporter | 2.19 |
| *lmo2306* | *lmo2306* | hypothetical protein | 4.07 |
| *lmo1375* | *lmo1375* | aminotripeptidase | 1.73 |
| *lmo0635* | *lmo0635* | hypothetical protein | -2.36 |
| *lmo1457* | *lmo1457* | hypothetical protein | -1.69 |
| *lmo2562* | *lmo2562* | hypothetical protein | -1.71 |
| *lmo1882* | *rpsN* | 30S ribosomal protein S14 | 2.20 |
| *lmo2700* | *lmo2700* | aldo/keto reductase | -1.87 |
| *lmo1012* | *lmo1012* | N-acyl-L-amino acid amidohydrolase | -2.02 |
| *lmo0487* | *lmo0487* | hypothetical protein | -1.64 |
| *lmo2768* | *lmo2768* | hypothetical protein | -2.42 |
| *lmo0957* | *lmo0957* | glucosamine-6-phosphate isomerase | 1.73 |
| *lmo1390* | *lmo1390* | ABC transporter permease | -1.75 |
| *lmo0194* | *lmo0194* | ABC transporter, ATP-binding protein | 1.79 |
| *lmo1731* | *lmo1731* | sugar ABC transporter permease | 3.30 |
| *lmo0791* | *lmo0791* | hypothetical protein | -1.66 |
| *lmo1027* | *lmo1027* | hypothetical protein | 2.03 |
| *lmo0981* | *lmo0981* | transporter | -1.78 |
| *lmo1960* | *fhuC* | ferrichrome ABC transporter ATP-binding protein | -3.46 |
| *lmo2575* | *lmo2575* | cation transporter | 2.18 |
| *lmo1660* | *leuS* | leucyl-tRNA synthetase EC_Number 6.1.1.4 | -1.53 |
| *lmo0829* | *nifJ* | pyruvate-flavodoxin oxidoreductase | -1.59 |
| *lmo1482* | *comEC* | competence protein ComEC | 2.31 |
| *lmo0824* | *lmo0824* | hypothetical protein | -2.15 |
| *lmo1963* | *lmo1963* | hypothetical protein | 2.54 |
| *lmo1031* | *lmo1031* | hypothetical protein | 2.75 |
| *lmo0958* | *lmo0958* | GntR family transcirptional regulator | 2.33 |
| *lmo0478* | *lmo0478* | secreted protein | -3.18 |
| *lmo1336* | *lmo1336* | 5-formyltetrahydrofolate cyclo-ligase | -1.84 |
| *lmo0411* | *lmo0411* | phosphoenolpyruvate synthase | 1.92 |
| *lmo0961* | *lmo0961* | protease | -2.41 |
| *lmo2468* | *clpP* | ATP-dependent Clp protease proteolytic subunit EC_Number 3.4.21.92 | 2.29 |
| *lmo0227* | *lmo0227* | hypothetical protein | -1.65 |
| *lmo1335* | *rpmG* | 50S ribosomal protein L33 | -2.65 |
| *lmo1866* | *lmo1866* | hypothetical protein | 1.79 |
| *lmo0230* | *lmo0230* | hypothetical protein | 2.50 |
| *lmo1033* | *lmo1033* | transketolase | 2.73 |
| *lmo1082* | *lmo1082* | dTDP-sugar epimerase | -1.65 |
| *lmo0163* | *lmo0163* | hypothetical protein | -1.40 |
| *lmo2181* | *srtB* | sortase B | -3.60 |
| *lmo0092* | *lmo0092* | ATP synthase F0F1 subunit beta EC_Number 3.6.3.14 | 1.95 |
| *lmo0198* | *glmU* | bifunctional N-acetylglucosamine-1-phosphate uridyltransferase/glucosamine-1-phosphate acetyltransferase | -1.52 |
| *lmo1473* | *dnaK* | molecular chaperone DnaK heat shock protein 70 | 2.81 |
| *lmo2299* | *lmo2299* | portal protein portal protein [Bacteriophage A118] | 2.43 |
| *lmo1475* | *hrcA* | heat-inducible transcription repressor | 2.84 |
| *lmo2325a* | *lmo2325a* | hypothetical protein | -3.12 |
| *lmo2458* | *pgk* | phosphoglycerate kinase EC_Number 2.7.2.3 | -1.92 |
| *lmo0445* | *lmo0445* | transcripitonal regulator | 2.38 |
| *lmo1544* | *minD* | septum formation inhibitor MinD | -1.53 |
| *lmo1238* | *rph* | ribonuclease PH EC_Number 2.7.7.56 | -1.53 |
| *lmo1880* | *lmo1880* | RNase HI | 1.44 |
| *lmo1020* | *lmo1020* | hypothetical protein | 1.64 |
| *lmo1232* | *lmo1232* | recombination and DNA strand exchange inhibitor protein MutS2 | 1.56 |
| *lmo1465* | *lmo1465* | metalloprotease | -1.83 |
| *lmo1526* | *lmo1526* | hypothetical protein | 2.68 |
| *lmo2057* | *ctaB* | protoheme IX farnesyltransferase | 1.39 |
| *lmo0786* | *lmo0786* | ACP phosphodiesterase | -1.70 |
| *lmo0335* | *lmo0335* | hypothetical protein | 2.87 |
| *lmo1851* | *lmo1851* | carboxy-terminal processing proteinase | -1.67 |
| *lmo0114* | *lmo0114* | repressor C1 | 1.71 |
| *lmo0286* | *lmo0286* | aminotransferase | -1.70 |
| *lmo2079* | *lmo2079* | hypothetical protein | -1.80 |
| *lmo2170* | *lmo2170* | hypothetical protein | 2.34 |
| *lmo0739* | *lmo0739* | 6-phospho-beta-glucosidase | 2.15 |
| *lmo1300* | *lmo1300* | arsenic transporter | 1.40 |
| *lmo0435* | *lmo0435* | peptidoglycan binding protein | 1.59 |
| *lmo1919* | *lmo1919* | hypothetical protein | 1.79 |
| *lmo0097* | *lmo0097* | PTS mannose transporter subunit IIC | -1.76 |
| *lmo0495* | *lmo0495* | hypothetical protein | 1.79 |
| *lmo2462* | *lmo2462* | dipeptidase | 1.41 |
| *lmo1883* | *lmo1883* | chitinase | 2.57 |
| *lmo0039* | *lmo0039* | carbamate kinase EC_Number 2.7.2.2 | 1.97 |
| *lmo1555* | *hemD* | uroporphyrinogen-III synthase EC_Number 4.2.1.75 | -2.37 |
| *lmo1849* | *lmo1849* | metal ABC transporter ATP-binding protein | 1.82 |
| *lmo2409* | *lmo2409* | hypothetical protein | -3.32 |
| *lmo2551* | *rho* | transcription termination factor Rho | -1.84 |
| *lmo1539* | *lmo1539* | glycerol transporter | 2.13 |
| *lmo0113* | *lmo0113* | hypothetical protein | 2.61 |
| *lmo2347* | *lmo2347* | amino acid ABC transporter permease | -3.07 |
| *lmo2142* | *lmo2142* | hypothetical protein | -1.86 |
| *lmo2213* | *lmo2213* | hypothetical protein | 2.79 |
| *lmo2277* | *lmo2277* | hypothetical protein | -2.15 |
| *lmo1958* | *fhuB* | ferrichrome ABC transporter permease | -3.13 |
| *lmo2378* | *lmo2378* | monovalent cation/H+ antiporter subunit A | 1.30 |
| *lmo0383* | *lmo0383* | methylmalonate-semialdehyde dehydrogenase | 2.46 |
| *lmo2638* | *lmo2638* | NADH dehydrogenase | -1.49 |
| *lmo2348* | *lmo2348* | amino acid ABC transporter permease | -2.44 |
| *lmo0781* | *lmo0781* | PTS mannose transporter subunit IID | 2.41 |
| *lmo0025* | *lmo0025* | phosphoheptose isomerase | 2.19 |
| *lmo1991* | *ilvA* | threonine dehydratase EC_Number 4.3.1.19 | 1.54 |
| *lmo0177* | *metS* | methionyl-tRNA synthetase EC_Number 6.1.1.10 | -1.36 |
| *lmo2350* | *lmo2350* | hypothetical protein | -2.58 |
| *lmo2115* | *lmo2115* | ABC transporter permease | 2.31 |
| *lmo1304* | *lmo1304* | hypothetical protein | 1.46 |
| *lmo0447* | *lmo0447* | glutamate decarboxylase | 2.78 |
| *lmo1340* | *lmo1340* | hypothetical protein | 1.93 |
| *lmo1961* | *lmo1961* | oxidoreductase | -2.91 |
| *lmo0724* | *lmo0724* | hypothetical protein | 2.26 |
| *lmo1533* | *ruvA* | Holliday junction DNA helicase RuvA | -1.49 |
| *lmo1730* | *lmo1730* | sugar ABC transporter substrate-binding protein | 2.48 |
| *lmo2754* | *lmo2754* | D-alanyl-D-alanine carboxypeptidase | -1.65 |
| *lmo2154* | *nrdF* | ribonucleotide-diphosphate reductase subunit beta EC_Number 1.17.4.1 | -1.67 |
| *lmo0099* | *lmo0099* | hypothetical protein | -1.52 |
| *lmo0537* | *lmo0537* | allantoate amidohydrolase | -2.20 |
| *lmo0185* | *lmo0185* | hypothetical protein | -1.39 |
| *lmo0666* | *lmo0666* | hypothetical protein | -2.08 |
| *lmo2602* | *lmo2602* | hypothetical protein | 2.38 |
| *lmo1239* | *lmo1239* | nucleoside-triphosphatase | -1.40 |
| *lmo2725* | *lmo2725* | hypothetical protein | 1.56 |
| *lmo0090* | *lmo0090* | ATP synthase F0F1 subunit alpha EC_Number 3.6.3.14 | 2.27 |
| *lmo1915* | *lmo1915* | malate dehydrogenase malic enzyme | -1.57 |
| *lmo2698* | *lmo2698* | RpiR family transcriptional regulator | 1.75 |
| *lmo2712* | *lmo2712* | gluconate kinase | -1.63 |
| *lmo0098* | *lmo0098* | PTS mannose transporter subunit IID | -1.42 |
| *lmo2440* | *lmo2440* | hypothetical protein | -2.62 |
| *lmo1373* | *lmo1373* | branched-chain alpha-keto acid dehydrogenase subunit E1 | -1.69 |
| *lmo1804* | *smc* | chromosome condensation protein Smc | -1.87 |
| *lmo0737* | *lmo0737* | hypothetical protein | 2.92 |
| *lmo1602* | *lmo1602* | hypothetical protein | 1.41 |
| *lmo2191* | *spxA* | ArsC family transcriptional regulator | 1.96 |
| *lmo1867* | *lmo1867* | pyruvate phosphate dikinase EC_Number 2.7.9.1 | 1.61 |
| *lmo1937* | *engA* | GTP-binding protein EngA | -1.64 |
| *lmo0845* | *lmo0845* | hypothetical protein | -1.58 |
| *lmo2721* | *lmo2721* | 6-phosphogluconolactonase | 1.85 |
| *lmo1557* | *hemA* | glutamyl-tRNA reductase | -1.79 |
| *lmo2456* | *pgm* | phosphoglyceromutase | -2.06 |
| *lmo0103* | *lmo0103* | NADH oxidase | 1.74 |
| *lmo1819* | *lmo1819* | ribosome-associated GTPase EngCs | -1.34 |
| *lmo0773* | *lmo0773* | alcohol dehydrogenase | -1.72 |
| *lmo0948* | *lmo0948* | transcriptional regulator | 1.59 |
| *lmo0847* | *lmo0847* | glutamine ABC transporter | -2.24 |
| *lmo0587* | *lmo0587* | secreted protein | 1.71 |
| *lmo1949* | *lmo1949* | hypothetical protein | -1.58 |
| *lmo1664* | *metK* | S-adenosylmethionine synthetase EC_Number 2.5.1.6 | -1.77 |
| *lmo0995* | *lmo0995* | hypothetical protein | 2.75 |
| *lmo1237* | *racE* | glutamate racemase | -1.64 |
| *lmo0844* | *lmo0844* | hypothetical protein | -1.72 |
| *lmo1359* | *nusB* | transcription antitermination protein NusB | -1.44 |
| *lmo1426* | *opuCC* | glycine/betaine ABC transporter substrate-binding protein | 1.91 |
| *lmo2767* | *lmo2767* | hypothetical protein | -1.63 |
| *lmo2651* | *lmo2651* | PTS mannitol transporter subunit IIA | 2.74 |
| *lmo2273* | *lmo2273* | hypothetical protein | 2.09 |
| *lmo2758* | *guaB* | inosine-monophosphate dehydrogenase | -1.83 |
| *lmo0277* | *lmo0277* | oxidoreductase | -2.07 |
| *lmo0732* | *lmo0732* | peptidoglycan binding protein | 1.89 |
| *lmo0870* | *lmo0870* | hypothetical protein | 2.11 |
| *lmo1809* | *plsX* | glycerol-3-phosphate acyltransferase PlsX | -1.47 |
| *lmo2457* | *tpiA* | triosephosphate isomerase | -1.72 |
| *lmo2219* | *prsA2* | foldase | 2.11 |
| *lmo1705* | *lmo1705* | deoxyguanosine kinase/deoxyadenosine kinase | -1.81 |
| *lmo2198* | *trpS* | tryptophanyl-tRNA synthetase EC_Number 6.1.1.2 | -1.67 |
| *lmo1784* | *rpmI* | 50S ribosomal protein L35 | 1.74 |
| *lmo2434* | *lmo2434* | glutamate decarboxylase | 2.46 |
| *lmo1114* | *lmo1114* | hypothetical protein | 2.34 |
| *lmo1112* | *lmo1112* | hypothetical protein | 2.47 |
| *lmo1589* | *argB* | acetylglutamate kinase EC_Number 2.7.2.8 | -3.10 |
| *lmo1133* | *lmo1133* | hypothetical protein | 3.54 |
| *lmo0369* | *lmo0369* | hypothetical protein | -1.35 |
| *lmo1444* | *lmo1444* | foldase | 2.18 |
| *lmo2067* | *lmo2067* | bile acid hydrolase | 2.58 |
| *lmo1559* | *thrS* | threonyl-tRNA synthetase | -1.37 |
| *lmo1857* | *lmo1857* | hypothetical protein | -1.82 |
| *lmo0777* | *lmo0777* | hypothetical protein | -1.40 |
| *lmo1914* | *lmo1914* | hypothetical protein | -1.40 |
| *lmo2459* | *gap* | glyceraldehyde-3-phosphate dehydrogenase | -1.97 |
| *lmo0819* | *lmo0819* | hypothetical protein | 2.18 |
| *lmo1228* | *rnhC* | ribonuclease HIII EC_Number 3.1.26.4 | -1.42 |
| *lmo2428* | *lmo2428* | cell division protein FtsW | -1.29 |
| *lmo2180* | *lmo2180* | hypothetical protein | -3.21 |
| *lmo1729* | *bglX* | beta-glucosidase | 1.92 |
| *lmo1575* | *lmo1575* | hypothetical protein | 1.39 |
| *lmo2202* | *fabH* | 3-oxoacyl-ACP synthase EC_Number 2.3.1.41 | -2.01 |
| *lmo1471* | *prmA* | ribosomal protein L11 methyltransferase | 1.54 |
| *lmo1734* | *lmo1734* | glutamate synthase large subunit | 1.93 |
| *lmo1518* | *lmo1518* | hypothetical protein | 1.98 |
| *lmo1306* | *lmo1306* | hypothetical protein | -1.42 |
| *lmo2030* | *lmo2030* | hypothetical protein | 1.42 |
| *lmo2355* | *lmo2355* | multidrug resistance protein | -1.51 |
| *lmo2516* | *lmo2516* | hypothetical protein | -1.46 |
| *lmo2153* | *lmo2153* | flavodoxin An electron-transfer protein | -1.62 |
| *lmo1328* | *truB* | tRNA pseudouridine synthase B | -1.83 |
| *lmo1350* | *lmo1350* | glycine dehydrogenase subunit 2 EC_Number 1.4.4.2 | 1.70 |
| *lmo1248* | *lmo1248* | hypothetical protein | -1.72 |
| *lmo2436* | *lmo2436* | transcription antiterminator | 1.63 |
| *lmo1818* | *rpe-2* | ribulose-phosphate 3-epimerase EC_Number 5.1.3.1 | -1.67 |
| *lmo0867* | *lmo0867* | hypothetical protein | -1.92 |
| *lmo0816* | *lmo0816* | regulatory protein PaiA | 1.95 |
| *lmo0848* | *lmo0848* | amino acid ABC transporter ATP-binding protein | -1.74 |
| *lmo0026* | *lmo0026* | copper homeostasis protein CutC | 2.64 |
| *lmo1916* | *lmo1916* | peptidase | -1.37 |
| *lmo1830* | *lmo1830* | short-chain dehydrogenase | 2.07 |
| *lmo1903* | *lmo1903* | thioredoxin | -1.64 |
| *lmo1663* | *ansB* | asparagine synthetase | -1.41 |
| *lmo2053* | *lmo2053* | hypothetical protein | -1.55 |
| *lmo0022* | *lmo0022* | PTS fructose transporter subunit IIB | 3.58 |
| *lmo1041* | *lmo1041* | molybdate ABC transporter substrate-binding protein | -2.24 |
| *lmo0134* | *lmo0134* | hypothetical protein | 2.10 |
| *lmo2739* | *lmo2739* | NAD-dependent deacetylase | 1.60 |
| *lmo0872* | *lmo0872* | antibiotic resistance protein | 1.73 |
| *lmo0003* | *lmo0003* | hypothetical protein | -1.81 |
| *lmo1106* | *lmo1106* | hypothetical protein | 2.36 |
| *lmo2371* | *lmo2371* | ABC transporter permease | -1.98 |
| *lmo0355* | *lmo0355* | fumarate reductase subunit A EC_Number 1.3.1.6 | -2.38 |
| *lmo1646* | *lmo1646* | exonuclease SbcD | -1.65 |
| *lmo0782* | *lmo0782* | PTS mannose transporter subunit IIC | 2.18 |
| *lmo0038* | *lmo0038* | agmatine deiminase | 1.65 |
| *lmo1007* | *lmo1007* | hypothetical protein | -2.97 |
| *lmo1118* | *lmo1118* | hypothetical protein | -1.33 |
| *lmo1803* | *lmo1803* | cell division protein FtsY | -1.59 |
| *lmo1981* | *lmo1981* | hypothetical protein | -1.52 |
| *lmo1186* | *lmo1186* | ethanolamine utilization protein EutH | 2.26 |
| *lmo2718* | *cydA* | cytochrome D ubiquinol oxidase subunit I | -1.83 |
| *lmo2111* | *lmo2111* | nitroreductase | -1.70 |
| *lmo0669* | *lmo0669* | oxidoreductase | 2.11 |
| *lmo2068* | *groEL* | molecular chaperone GroEL 60 kDa chaperone family | 2.40 |
| *lmo0508* | *lmo0508* | PTS galactitol transporter subunit IIC | 1.82 |
| *lmo0690* | *flaA* | flagellin structural flagella protein | 2.19 |
| *lmo0895* | *sigB* | RNA polymerase sigma factor SigB | 1.28 |
| *lmo1724* | *lmo1724* | ABC transporter ATP-binding protein | 2.21 |
| *lmo1837* | *pyrC* | dihydroorotase EC_Number 3.5.2.3 | -2.74 |
| *lmo1858* | *lmo1858* | dehydrogenase | -1.69 |
| *lmo2536* | *atpI* | ATP synthase subunit I | -1.40 |
| *lmo0245* | *secE* | preprotein translocase subunit SecE | -1.55 |
| *lmo0331* | *lmo0331* | internalin | 1.52 |
| *lmo1423* | *lmo1423* | hypothetical protein | 1.35 |
| *lmo2383* | *lmo2383* | monovalent cation/H+ antiporter subunit F | 1.55 |
| *lmo0288* | *lmo0288* | two-component sensor histidine kinase | -1.13 |
| *lmo1790* | *lmo1790* | hypothetical protein | 1.96 |
| *lmo0272* | *lmo0272* | hypothetical protein | -1.26 |
| *lmo1545* | *minC* | septum formation inhibitor MinC | -1.52 |
| *lmo2196* | *oppA* | hypothetical protein | -1.66 |
| *lmo0384* | *lmo0384* | IolB protein | 2.73 |
| *lmo0470* | *lmo0470* | hypothetical protein | -1.47 |
| *lmo1251* | *lmo1251* | Fnr/Crp family transcriptional regulator | -2.15 |
| *lmo1481* | *holA* | DNA polymerase III subunit delta | 1.25 |
| *lmo1957* | *fhuG* | ferrichrome ABC transporter permease | -2.43 |
| *lmo2588* | *lmo2588* | multidrug transporter | 1.26 |
| *lmo1717* | *lmo1717* | hypothetical protein | -2.14 |
| *lmo0219* | *lmo0219* | hypothetical protein | -1.14 |
| *lmo1795* | *lmo1795* | hypothetical protein | 1.12 |
| *lmo0589* | *lmo0589* | hypothetical protein | 1.66 |
| *lmo2227* | *lmo2227* | ABC transporter ATP-binding protein | 2.07 |
| *lmo0485* | *lmo0485* | hypothetical protein | -2.30 |
| *lmo1964* | *lmo1964* | ABC transporter ATP-binding protein | 1.90 |
| *lmo0162* | *holB* | DNA polymerase III subunit delta' EC_Number 2.7.7.7 | -1.27 |
| *lmo0788* | *lmo0788* | hypothetical protein | -2.25 |
| *lmo1698* | *lmo1698* | ribosomal-protein-alanine N-acetyltransferase | 2.20 |
| *lmo1972* | *lmo1972* | PTS pentitol transporter subunit IIB | 2.92 |
| *lmo0473* | *lmo0473* | hypothetical protein | -1.79 |
| *lmo1034* | *lmo1034* | glycerol kinase | 2.45 |
| *lmo2450* | *lmo2450* | carboxylesterase | 1.35 |
| *lmo0418* | *lmo0418* | hypothetical protein | -1.77 |
| *lmo2646* | *lmo2646* | hypothetical protein | 2.55 |
| *lmo0969* | *lmo0969* | ribosomal large subunit pseudouridine synthetase | -1.69 |
| *lmo0674* | *mogR* | hypothetical protein | 1.79 |
| *lmo1378* | *lisK* | two-component sensor histidine kinase | 1.24 |
| *lmo2465* | *lmo2465* | hypothetical protein | -1.39 |
| *lmo2587* | *lmo2587* | hypothetical protein | -1.91 |
| *lmo2636* | *lmo2636* | hypothetical protein | -1.26 |
| *lmo0538* | *lmo0538* | N-acyl-L-amino acid amidohydrolase | -1.74 |
| *lmo2431* | *lmo2431* | ferrichrome ABC transporter substrate-binding protein | -1.61 |
| *lmo1764* | *purD* | phosphoribosylamine--glycine ligase | 1.76 |
| *lmo2577* | *lmo2577* | hypothetical protein | -1.41 |
| *lmo0979* | *lmo0979* | daunorubicin resistance ATP-binding protein | 2.11 |
| *lmo1474* | *grpE* | heat shock protein GrpE | 2.27 |
| *lmo2200* | *lmo2200* | MarR family transcriptional regulator | 1.56 |
| *lmo2784* | *lmo2784* | transcriptional antiterminator | 1.23 |
| *lmo2441* | *lmo2441* | transcriptional regulator | -1.63 |
| *lmo2568* | *lmo2568* | hypothetical protein | 2.38 |
| *lmo1079* | *lmo1079* | hypothetical protein | -1.34 |
| *lmo2835* | *lmo2835* | xylose isomerase | 2.57 |
| *lmo1552* | *valS* | valyl-tRNA synthetase EC_Number 6.1.1.9 | -1.40 |
| *lmo2644a* | *lmo2644a* | hypothetical protein | 2.35 |
| *lmo2021* | *lmo2021* | hypothetical protein | -1.73 |
| *lmo2307* | *lmo2307* | hypothetical protein | 3.21 |
| *lmo2130* | *lmo2130* | hypothetical protein | 1.19 |
| *lmo1911* | *lmo1911* | histidine kinase | -1.28 |
| *lmo2573* | *lmo2573* | zinc-binding dehydrogenase | 2.09 |
| *lmo1285* | *lmo1285* | hypothetical protein | 1.20 |
| *lmo0613* | *lmo0613* | oxidoreductase | 1.63 |
| *lmo0347* | *lmo0347* | dihydroxyacetone kinase | 2.18 |
| *lmo0758* | *lmo0758* | hypothetical protein | 1.73 |
| *lmo0021* | *lmo0021* | PTS fructose transporter subunit IIA | 2.65 |
| *lmo0572* | *lmo0572* | hypothetical protein | -1.53 |
| *lmo0890* | *rsbS* | negative regulation of sigma-B activity | -1.80 |
| *lmo2530* | *atpG* | ATP synthase F0F1 subunit gamma | -1.09 |
| *lmo1591* | *argC* | N-acetyl-gamma-glutamyl-phosphate reductase EC_Number 1.2.1.38 | -2.34 |
| *lmo1235* | *lmo1235* | aspartate kinase EC_Number 2.7.2.4 | -1.79 |
| *lmo0696* | *flgD* | flagellar basal body rod modification protein | -2.39 |
| *lmo1436* | *lmo1436* | aspartate kinase EC_Number 2.7.2.4 | -1.15 |
| *lmo1553* | *hemL* | glutamate-1-semialdehyde aminotransferase EC_Number 5.4.3.8 | -1.38 |
| *lmo1587* | *argF* | ornithine carbamoyltransferase | -2.39 |
| *lmo2367* | *pgi* | glucose-6-phosphate isomerase EC_Number 5.3.1.9 | -1.52 |
| *lmo1438* | *lmo1438* | penicillin-binding protein | 1.32 |
| *lmo1072* | *pycA* | pyruvate carboxylase EC_Number 6.4.1.1 | -1.09 |
| *lmo0614* | *lmo0614* | hypothetical protein | -1.92 |
| *lmo2139* | *lmo2139* | ABC transporter ATP-binding protein | -1.23 |
| *lmo0596* | *lmo0596* | hypothetical protein | 2.20 |
| *lmo2696* | *lmo2696* | dihydroxyacetone kinase | 2.08 |
| *lmo1792* | *trmD* | tRNA (guanine-N(1)-)-methyltransferase | 1.38 |
| *lmo0242* | *lmo0242* | hypothetical protein | -1.64 |
| *lmo0612* | *lmo0612* | MarR family transcriptional evidence | 1.47 |
| *lmo1746* | *lmo1746* | ABC transporter permease | 1.48 |
| *lmo0448* | *lmo0448* | amino acid antiporter | 2.41 |
| *lmo2744* | *lmo2744* | Crp/Fnr family transcriptional regulator | -1.53 |
| *lmo0837* | *lmo0837* | hypothetical protein | -1.81 |
| *lmo1025* | *lmo1025* | hypothetical protein | -1.66 |
| *lmo2108* | *lmo2108* | N-acetylglucosamine-6-phosphate deacetylase | 1.36 |
| *lmo2455* | *eno* | phosphopyruvate hydratase enolase | -1.77 |
| *lmo0894* | *rsbW* | serine-protein kinase RsbW EC_Number 2.7.11.1 | 1.28 |
| *lmo0891* | *rsbT* | positive regulation of sigma-B activity | -1.55 |
| *lmo0409* | *lmo0409* | internalin | 2.01 |
| *lmo0628* | *lmo0628* | hypothetical protein | 2.02 |
| *lmo0793* | *lmo0793* | hypothetical protein | -1.60 |
| *lmo1850* | *lmo1850* | MarR family transcriptional regulator | -1.19 |
| *lmo2773* | *lmo2773* | transcriptional antiterminator | 1.52 |
| *lmo1617* | *lmo1617* | multidrug transporter | 1.58 |
| *lmo1839* | *pyrP* | uracil permease | -2.61 |
| *lmo1908* | *lmo1908* | hypothetical protein | -1.38 |
| *lmo2750* | *lmo2750* | para-aminobenzoate synthase subunit I | -1.29 |
| *lmo2477* | *galE* | UDP-glucose 4-epimerase | -1.21 |
| *lmo1497* | *udk* | uridine kinase EC_Number 2.7.1.48 | -1.72 |
| *lmo1920* | *lmo1920* | hypothetical protein | -1.95 |
| *lmo1946* | *lmo1946* | acyl-CoA hydrolase | -1.26 |
| *lmo0218* | *lmo0218* | hypothetical protein | -1.34 |
| *lmo2275* | *lmo2275* | protein gp28 Portein gp28 [Bacteriophage A118] | 1.69 |
| *lmo0546* | *lmo0546* | NAD(P)-dependent oxidoreductase | 1.92 |
| *lmo1817* | *lmo1817* | hypothetical protein | -1.87 |
| *lmo1789* | *lmo1789* | hypothetical protein | 1.84 |
| *lmo0570* | *hisJ* | histidinol-phosphatase EC_Number 3.1.3.15 | -1.55 |
| *lmo1674* | *lmo1674* | prolyl aminopetidase | -1.42 |
| *lmo0458* | *lmo0458* | hydantoinase | 1.49 |
| *lmo0761* | *lmo0761* | hypothetical protein | 1.71 |
| *lmo2302* | *lmo2302* | hypothetical protein | 2.20 |
| *lmo2569* | *lmo2569* | peptide ABC transporter substrate-binding protein | -1.47 |
| *lmo1701* | *lmo1701* | hypothetical protein | 1.57 |
| *lmo1762* | *lmo1762* | hypothetical protein | 1.80 |
| *lmo0229* | *lmo0229* | CtsR family transcriptional regulator | 1.86 |
| *lmo2199* | *lmo2199* | hypothetical protein | 1.34 |
| *lmo0605* | *lmo0605* | hypothetical protein | -1.60 |
| *lmo1240* | *lmo1240* | hypothetical protein | -1.29 |
| *lmo1732* | *lmo1732* | sugar ABC transporter permease | 1.88 |
| *lmo0244* | *rpmG* | 50S ribosomal protein L33 type II | -1.53 |
| *lmo1716* | *lmo1716* | transcriptional regulator | 1.63 |
| *lmo0869* | *lmo0869* | hypothetical protein | 1.57 |
| *lmo1756* | *gatC* | aspartyl/glutamyl-tRNA amidotransferase subunit C | -1.29 |
| *lmo0269* | *lmo0269* | transporter | -1.26 |
| *lmo1064* | *lmo1064* | transporter | -1.22 |
| *lmo1840* | *pyrR* | bifunctional pyrimidine regulatory protein PyrR uracil phosphoribosyltransferase EC_Number 2.4.2.9 | -1.90 |
| *lmo2493* | *lmo2493* | ArsR family transcriptional regulator | 1.53 |
| *lmo1210* | *lmo1210* | hypothetical protein | -1.55 |
| *lmo1360* | *folD* | bifunctional 5,10-methylene-tetrahydrofolate dehydrogenase/ 5,10-methylene-tetrahydrofolate cyclohydrolase | -1.46 |
| *lmo2073* | *lmo2073* | ABC transporter ATP-binding protein | -1.23 |
| *lmo2605* | *rplQ* | 50S ribosomal protein L17 | 1.31 |
| *lmo0346* | *lmo0346* | triosephosphate isomerase | 2.26 |
| *lmo2063* | *lmo2063* | hypothetical protein | -2.44 |
| *lmo0831* | *lmo0831* | hypothetical protein | -1.36 |
| *lmo1543* | *lmo1543* | ribonuclease G | -1.35 |
| *lmo1951* | *scpA* | segregation and condensation protein A | -1.54 |
| *lmo2127* | *lmo2127* | hypothetical protein | -1.80 |
| *lmo1211* | *lmo1211* | hypothetical protein | -1.94 |
| *lmo2500* | *phoR* | two-component sensor histidine kinase | -1.42 |
| *lmo1977* | *lmo1977* | hypothetical protein | -1.56 |
| *lmo1371* | *lmo1371* | dihydrolipoamide dehydrogenase EC_Number 1.8.1.4 | -1.57 |
| *lmo0618* | *lmo0618* | protein kinase | -1.39 |
| *lmo0402* | *lmo0402* | transcriptional antiterminator BglG | -1.18 |
| *lmo0040* | *lmo0040* | agmatine deiminase | -1.56 |
| *lmo1217* | *lmo1217* | endo-1,4-beta-glucanase and to aminopeptidase | -1.30 |
| *lmo2557* | *lmo2557* | lipid kinase | -1.09 |
| *lmo0155* | *lmo0155* | zinc ABC transporter permease | 2.18 |
| *lmo0353* | *lmo0353* | hypothetical protein | 1.64 |
| *lmo2853* | *lmo2853* | hypothetical protein | -1.04 |
| *lmo2563* | *lmo2563* | hypothetical protein | -1.46 |
| *lmo1728* | *lmo1728* | cellobiose phosphorylase | 1.74 |
| *lmo1976* | *lmo1976* | oxidoreductase | -1.29 |
| *lmo2105* | *lmo2105* | ferrous iron transport protein B | -2.34 |
| *lmo1645* | *lmo1645* | ATP-dependent dsDNA exonuclease SbcC | -1.42 |
| *lmo2855* | *rnpA* | ribonuclease P EC_Number 3.1.26.5 | -1.91 |
| *lmo0586* | *lmo0586* | hypothetical protein | 1.92 |
| *lmo2019* | *ileS* | isoleucyl-tRNA synthetase EC_Number 6.1.1.5 | -1.16 |
| *lmo0024* | *lmo0024* | PTS mannose transporter subunit IID | 2.69 |
| *lmo0530* | *lmo0530* | hypothetical protein | -1.02 |
| *lmo1084* | *lmo1084* | DTDP-L-rhamnose synthetase | -1.09 |
| *lmo1993* | *pdp* | pyrimidine-nucleoside phosphorylase EC_Number 2.4.2.2 | -1.40 |
| *lmo0036* | *lmo0036* | putrescine carbamoyltransferase EC_Number 2.1.3.3 | 1.74 |

**Table S3** Genes with putative intracellular antisense regulation. Fold changes refer to antisense transcripts

| **Locus tag** | **Gene** | **Description** | | **log2FC** | |
| --- | --- | --- | --- | --- | --- |
| *lmo1136* | *lmo1136* | internalin | 5.56 | |  |
| *lmo1705* | *lmo1705* | deoxyguanosine kinase/deoxyadenosine kinase | 3.70 | |  |
| *lmo2679* | *lmo2679* | histidine kinase | 3.46 | |  |
| *lmo0996* | *lmo0996* | methylated-DNA-protein-cysteine methyltransferase | 4.34 | |  |
| *lmo2681* | *kdpB* | potassium-transporting ATPase subunit B EC_Number 3.6.3.12 | 4.75 | |  |
| *lmo1937* | *engA* | GTP-binding protein EngA | 4.36 | |  |
| *lmo0536* | *lmo0536* | 6-phospho-beta-glucosidase | 3.82 | |  |
| *lmo0521* | *lmo0521* | 6-phospho-beta-glucosidase | 3.62 | |  |
| *lmo0936* | *lmo0936* | nitroflavin reductase | 3.44 | |  |
| *lmo1961* | *lmo1961* | oxidoreductase | 4.03 | |  |
| *lmo2178* | *lmo2178* | peptidoglycan binding protein | 3.18 | |  |
| *lmo0934* | *lmo0934* | hypothetical protein | 3.16 | |  |
| *lmo0537* | *lmo0537* | allantoate amidohydrolase | 3.18 | |  |
| *lmo0199* | *prs* | ribose-phosphate pyrophosphokinase | 2.92 | |  |
| *lmo2680* | *kdpC* | potassium-transporting ATPase subunit C | 3.81 | |  |
| *lmo0209* | *lmo0209* | hypothetical protein | 4.06 | |  |
| *lmo0839* | *lmo0839* | tetracycline resistance protein | 2.92 | |  |
| *lmo2270* | *comK'* | competence protein ComK | 3.16 | |  |
| *lmo0595* | *lmo0595* | O-acetylhomoserine sulfhydrylase | 4.09 | |  |
| *lmo0947* | *lmo0947* | hypothetical protein | 2.65 | |  |
| *lmo0208* | *lmo0208* | hypothetical protein | 3.89 | |  |
| *lmo0497* | *lmo0497* | sugar transferase | 3.22 | |  |
| *lmo1890* | *lmo1890* | hypothetical protein | 3.86 | |  |
| *lmo0538* | *lmo0538* | N-acyl-L-amino acid amidohydrolase | 2.47 | |  |
| *lmo2325* | *lmo2325* | hypothetical protein | -3.22 | |  |
| *lmo0645* | *lmo0645* | amino acid transporter | 3.37 | |  |
| *lmo2516* | *lmo2516* | hypothetical protein | 2.70 | |  |
| *lmo0581* | *lmo0581* | hypothetical protein | 2.57 | |  |
| *lmo2677* | *lmo2677* | esterase | 2.30 | |  |
| *lmo2678* | *lmo2678* | XRE family transcriptional regulator | 2.20 | |  |
| *lmo0198* | *glmU* | bifunctional N-acetylglucosamine-1-phosphate uridyltransferase/glucosamine-1-phosphate acetyltransferase | 2.61 | |  |
| *lmo0424* | *lmo0424* | glucose uptake protein | -2.23 | |  |

**Table S4** Additional information for Figure 5B. Pairwise comparison of different wild type and mutants after 4h of infection with correction for inoculation amount.

| Strain | Estimate | Std.Error | z-value | p-value unadj. | p-value  adj. fdr |
| --- | --- | --- | --- | --- | --- |
| wt vs. Δ*lmo1119* | 1.091 | 0.195 | 5.593 | 0 | 0 |
| wt vs. Δ*lmo2316* | 0.194 | 0.385 | 0.504 | 0.619 | 0.743 |
| wt vs. Δ*lmo1119*/*lmo2316* | 1.119 | 0.181 | 6.179 | 0 | 0 |
| Δ*lmo2316* vs. Δ*lmo1119* | 0.897 | 0.397 | 2.262 | 0.034 | 0.051 |
| Δ*lmo1119* vs. Δ*lmo1119*/*lmo2316* | 0.028 | 0.205 | 0.135 | 0.894 | 0.894 |
| Δ*lmo2316* vs. Δ*lmo1119*/*lmo2316* | 0.925 | 0.39 | 2.372 | 0.027 | 0.051 |
|  |  |  |  |  |  |
| wt = wild type   *L. monocytogenes* EGD-e |  |  |  |  |  |

**Table S5** Repetition of the survival experiments in macrophage shown in Figure 5B including a *prfA* deletion mutant strain (Chatterjee et al., 2006) as negative control. Experiments were carried out in quadruplicate and log2 transformed colony forming unit (CFU) values were corrected by inoculation amount and repetition as covariables. Pairwise comparisons are given for each repetition of the experiment.

|  | overall | | Repetition 1 | | Repetition 2 | | Repetition 3 | | Repetition 4 | |
| --- | --- | --- | --- | --- | --- | --- | --- | --- | --- | --- |
|  | Estimate | FDR | Estimate | FDR | Estimate | FDR | Estimate | FDR | Estimate | FDR |
| EGD-e - Δ*lmo2316* | 0.071 | 0.300 | -0.184 | 0.096 | 0.220 | 0.046 | 0.066 | 0.539 | 0.169 | 0.129 |
| EGD-e - Δ*lmo1119* | 1.159 | < 0.001 | 1.147 | < 0.001 | 1.127 | < 0.001 | 0.883 | < 0.001 | 1.458 | < 0.001 |
| EGD-e - Δ*lmo1119/lmo2316* | 1.270 | < 0.001 | 1.183 | < 0.001 | 1.294 | < 0.001 | 1.001 | < 0.001 | 1.575 | < 0.001 |
| EGD-e - Δ*prfA* | 5.649 | < 0.001 | 5.727 | < 0.001 | 5.627 | < 0.001 | 5.302 | < 0.001 | 5.936 | < 0.001 |
| Δ*lmo2316* - Δ*lmo1119* | 1.088 | < 0.001 | 1.331 | < 0.001 | 0.908 | < 0.001 | 0.817 | < 0.001 | 1.289 | < 0.001 |
| Δ*lmo2316* - Δ*lmo1119/lmo2316* | 1.199 | < 0.001 | 1.367 | < 0.001 | 1.074 | < 0.001 | 0.936 | < 0.001 | 1.406 | < 0.001 |
| Δ*lmo2316* - Δ*prfA* | 5.578 | < 0.001 | 5.911 | < 0.001 | 5.407 | < 0.001 | 5.236 | < 0.001 | 5.767 | < 0.001 |
| Δ*lmo1119* - Δ*lmo1119/lmo2316* | 0.111 | 0.700 | 0.036 | 0.736 | 0.167 | 0.121 | 0.118 | 0.298 | 0.117 | 0.273 |
| Δ*lmo1119* - Δ*prfA* | 4.491 | < 0.001 | 4.580 | < 0.001 | 4.499 | < 0.001 | 4.419 | < 0.001 | 4.478 | < 0.001 |
| Δlmo*1119/lmo2316* - Δ*prfA* | 4.379 | < 0.001 | 4.544 | < 0.001 | 4.333 | < 0.001 | 4.301 | < 0.001 | 4.361 | < 0.001 |
