# Supplementary material for: A detailed view of the intracellular transcriptome of Listeria monocytogenes in murine macrophages using RNA-seq
Source: Front Microbiol. 2015 Oct 30;6:1199. doi: 10.3389/fmicb.2015.01199 (PMC4627465; doi:10.3389/fmicb.2015.01199)
Supplement: Supplementary file 5 [file Supplemental_Text.DOCX]

**Supplemental text**

**Supplemental text 1** Construction of chromosomal deletion mutants Δ*lmo2316,* Δ*lmo1119* and Δ*lmo1119/lmo2316.*

Flanking regions of *lmo2316* or *lmo1119* were amplified by PCR using primer pairs 1 and 2 for the 5’ flanking region and 3 and 4 for the 3’ flanking region (**Table S1**). The resulting PCR products were fused in a second PCR reaction using primers 1 and 4 and the product was cloned into pCR2.1-TOPO (Invitrogen/Life technologies, [Carlsbad](https://www.google.de/search?biw=1536&bih=730&q=carlsbad+kalifornien&stick=H4sIAAAAAAAAAGOovnz8BQMDgwsHnxCXfq6-gUlVRUp8rhIHiF1kUp6npZWdbKWfX5SemJdZlViSmZ-HwrHKSE1MKSxNLCpJLSqO31i_nTn83iWDzg-KZmez447Mnm0AAMj3eFNhAAAA&sa=X&sqi=2&ved=0CIgBEJsTKAEwDmoVChMI7NWSxKnJxwIVSVgUCh17VQAM), CA; USA). For the generation of Δ*lmo1119* or Δ*lmo2316* the pCR2.1-TOPO vector containing the flanking region of the respective gene was digested with *BamH*I, *Xho*I and *Nco*I (Fermentas/[Thermo Fisher Scientific](https://en.wikipedia.org/wiki/Thermo_Fisher_Scientific), Waltham, MA, USA) and inserted into *BamH*I *and Sal*I (Fermentas/[Thermo Fisher Scientific](https://en.wikipedia.org/wiki/Thermo_Fisher_Scientific), Waltham, MA, USA) digested pAUL-A. pAUL-A::Δ*lmo1119* and pAUL-A::Δ*lmo2316,*were transformed separately into *E. coli* DH10β and were isolated, sequenced and subsequently electroporated into *L. monocytogenes* wild-type strain to produce single knockout mutants Δ*lmo1119* and Δ*lmo2316*. To generate the isogenic double mutant Δ*lmo1119/lmo2316,* the vector pAUL-A::Δ*lmo1119* was electroporated into strain Δ*lmo2316.* Gene replacement for single and double mutant creation was performed as previously described (Schaeferkordt, 1995). Each isogenic deletion mutant was confirmed by DNA sequencing of PCR products using primer 7 and 8 (**Table S1**).

**Reference**

Schäferkordt, S., and Chakraborty, T. (1995). Vector plasmid for insertional mutagenesis and directional cloning in *Listeria* spp. *Biotechniques*.19, 720-722, 724-725.

**Supplemental text 2** Differential gene expression operon analysis.

All operons were screened for the presence of differentially expressed genes. Among the 517 known operons, 316 did not contain differentially expressed genes. In 37 operons every single gene was upregulated under intracellular growth condition and 22 operons were uniformly downregulated compared to extracellular growth.

This adds up to 375 operons representing 72% of all operons. In that sense, nearly three quarter of all operons show perfect agreement with the expectation that genes that are most probably transcribed together also show similar patterns of expression.

For 139 operons (almost 27%) some but not all genes were found to be differentially expressed albeit all differentially regulated genes indicated a change in the same direction. An example is provided by the differential expression of *lmo1470-1475*, an operon primarily formed by class I heat shock genes. For this operon 4 out of 5 genes were significantly upregulated under intracellular growth conditions. The fifth gene (*lmo1470*) was considered not to be differentially expressed as the adjusted *p-*value of 0.095 exceeds the threshold.

Interestingly, for the remaining three operons, non-uniform directions of regulation were observed. As the expression patterns of these operons are in discordance with the proposed operon structure, a manual examination of read mappings was performed. The pattern of read mappings questions the proposed operon structure under intracellular growth conditions. **Figure S3** provides an overview of these operons. The *sigB* operon (*lmo0887-0896*) is the most remarkable among those. While *lmo0888* to *lmo0892* were downregulated during intracellular growth, *lmo0893* to *lmo0896* were found to be upregulated. This highlights that alternative mechanisms like transcription from an alternative promoter near *lmo0893* are most likely.
